# Adipose- and bone marrow-derived stromal cells reduce pain in patients with knee osteoarthritis but do not substantially improve knee functionality: an updated systematic review and meta-analysis

**DOI:** 10.1007/s00590-025-04322-4

**Published:** 2025-05-23

**Authors:** Erik Hohmann, Natalie Keough, Daniel Stokes, Rachel Frank, Scott Rodeo

**Affiliations:** 1 Medical School, Faculty of Health Sciences, University of Pretoria, Pretoria, South Africa; 2 University of Warwick, Coventry, UK; 3 University of Colorado Denver, Denver, US; 4 Hospital for Special Surgery, New York, US

**Keywords:** Adipose derived stem cells, Adipose derived stromal cells, Bone marrow derived stromal cells, Bone marrow derived stem cells, Knee osteoarthritis, Orthobiologics

## Abstract

**Purpose:**

To perform a systematic review and meta-analysis of randomized and comparative studies comparing mesenchymal stromal cells other orthobiological injections for patients with knee osteoarthritis.

**Methods:**

Systematic review of Medline, Embase, Scopus, and Google Scholar, including all level 1–3 from 2014 to 2024. Validated scores (VAS, KOOS, Lysholm, IKDC) were included as outcome measures. Risk of bias was assessed using the Cochrane Collaboration’s tools. The GRADE system was used to assess the quality of the body of evidence and the modified Coleman Methodology score was used to assess study quality. Heterogeneity was assessed using χ^2^ and I^2^ statistics.

**Results:**

Ten studies were included; all published in English between 2019 and 2023, encompassing a total of 563 cases (281 treated with MSCs and 282 with other biologics). Two studies had a high risk of bias, one had some bias, and seven had a low risk of bias. Publication bias was detected (Egger's test 3.26447; *p* = 0.007). The pooled estimates revealed significant differences favoring MSCs for VAS scores at 3, 6, and 12 months. For KOOS pain and symptoms, significant differences were observed at 3 and 6 months.

**Conclusion:**

The results of this meta-analysis demonstrated a significant effect of adipose and bone marrow-derived stromal cell injections on pain reduction at all assessed time points, and showed superiority over other non-surgical treatment options. These differences were not reflected in clinical and functional outcomes, indicating that the observed reduction in pain did not correspond to substantial improvements in knee functionality.

**Supplementary Information:**

The online version contains supplementary material available at 10.1007/s00590-025-04322-4.

## Introduction

Conservative treatment of osteoarthritis remains a challenging problem. The American College of Rheumatology/Arthritis Foundation Guidelines for the management of osteoarthritis of the hand, hip and knee provided strong recommendations for treatment [1]. They determined that exercise, weight loss, tibiofemoral bracing, topical nonsteroidal anti-inflammatory drugs, and intra-articular steroid injections are strongly supported by evidence [1]. Similarly, the 2019 OARSI guidelines for the non-surgical management of knee osteoarthritis recommend weight management, exercise programs, topical and systemic anti-inflammatory drugs, and intra-articular injections with hyaluronic acid and corticosteroids [2]. These first-line interventions are effective regardless of other variables such as age, sex, BMI, severity of osteoarthritis, and the presence of depression and other comorbidities [3].

The use of orthobiologics, regenerative therapies, and other intra-articular high molecular weight hyaluronic acid has gained substantial attention in recent years [4]. These therapies include platelet-rich plasma (PRP), stromal cells such as mesenchymal stromal cells (MSCs) and adipose-derived cells (ADSCs), bone marrow aspirate, stromal vascular fraction, micro-fragmented adipose tissue, and cells derived from peri-natal tissues, including umbilical cord blood, amniotic fluid, and placental-derived cells [4]. The principal mechanism of action of orthobiologics is that they primarily alleviate symptoms, likely due to their anti-inflammatory and immune-modulating effects, rather than inducing true tissue regeneration [4].

The application of intra-articular injections with MSCs has recently been proposed as a feasible alternative treatment option [5]. Several clinical studies involving MSCs failed to demonstrate functional improvement or pain relief compared to other orthobiologics and placebo [6–8]. In contrast, other publications showed that autologous MSCs led to significant pain relief and functional improvements at 6 months compared to placebo [9–11].

The purpose of this study was therefore to perform a systematic review and meta-analysis of randomized controlled and comparative observational studies comparing MSCs and other available orthobiological injections for patients with knee osteoarthritis.

## Material and methods

This study adhered to the Reporting Items for Systematic Reviews and Meta-Analysis (PRISMA) guidelines [12], along with the updated guidelines outlined in the Cochrane Handbook [13].

### Eligibility criteria

This project included studies comparing autologous and cultured adipose- and bone-marrow-derived stromal cells to other orthobiologic interventions including platelet-rich plasma (PRP), bone marrow aspirate concentrate (BMAC), synthetic agents like hyaluronic acid, corticosteroid injections, and placebos, provided they met specific inclusion and exclusion criteria. Level I–III evidence studies were eligible for inclusion if they were published between January 2014 and June 2024. Level III evidence studies were considered for inclusion to augment sample size and enhance generalizability of the results [14]. Previous research has indicated that incorporating Level III evidence studies does not alter the risk estimate of treatment effects derived from meta-analyses of randomized controlled trials, observational studies, or a combination of both [15–18]. Additionally, studies comparing more than two interventions were included in the analysis. For analysis, each intervention was then compared to the ADSC/BMSC treatment arm. 

Additional inclusion criteria comprised symptomatic knee osteoarthritis (Kellgren Grade I–IV), mean participant age exceeding 40 years, a minimum follow-up duration of 6 months, and inclusion of at least one validated outcome score (such as KOOS, VAS, IKDC, Lysholm). Studies involving patients who had previously received knee injections prior to study commencement or had these agents administered as adjuvant treatment with or without knee surgery within the past 6 months were excluded. Clinical case level of evidence (LOE) IV studies, case series, abstracts, and conference proceedings were likewise excluded. If the LOE was not specified in the manuscript, the level of evidence was determined as per standard research guidelines [19].

### Literature search

A systematic review of the literature was performed to identify all publications in English and German, screening the databases Medline, Embase, Scopus, and Google Scholar. These databases were screened using the following terms and Boolean operators: “orthobiologics” AND/OR “platelet-rich plasma” AND/OR “PRP” AND/OR “bone marrow aspirate concentrate”; AND/OR “BMAC” AND/OR “hyaluronic acid” AND/OR “adipose stem cells injections” AND/OR “adipose stromal cells injections”, AND/OR “knee osteoarthritis”; AND/OR “degenerative knee”. For the Medline search the MeSH term “osteoarthritis, knee” was used with the following qualifiers: drug therapy and therapy. One reviewer conducted independent title and abstract screening. Disagreements between reviewers were resolved by consensus, and if no consensus was reached, they were carried forward to the full-text review. All eligible articles were manually cross-referenced to ensure that other potential studies were identified.

### Data extraction and quality assessment

An electronic data extraction form was utilized to gather information from each article, including level of evidence, country, age, sex, length of follow-up, sample size, clinical outcome scores, range of motion, and complications. Risk of bias was assessed using the Cochrane Collaboration’s Risk of Bias Tool [13]. The GRADE system was used by three reviewers to assess the quality of the body of evidence for each outcome measure [13]. The recommendations from the Cochrane Handbook were followed, and an initial level of certainty was assigned. Studies were downgraded if there was a high risk of bias, inconsistency and imprecision of the results, and indirectness of evidence. Studies were upgraded if there were large treatment effects, a dose–response, or reasons to oppose plausible residual bias and confounding effects. Any disagreement between reviewers was resolved by consensus and/or by arbitration between the two senior authors. The modified Coleman Methodology Score was used as a second validated instrument to assess the quality of the included studies. The score ranges from 0 to 100 and the final score was categorized as excellent (85–100 points), good (70–84 points), fair (55–60 points), and poor (< 55 points) [20].

Inter-observer differences for study eligibility and risk of bias were measured using Cohen’s kappa coefficient. Heterogeneity of the data was assessed using χ2 and I^2^ statistics. Outcomes were pooled using a random effects model if the I^2^ statistic was > 25%, and a fixed model was used if the statistic was < 25%. Pooling of data for clinical outcomes was only performed if a minimum of three studies were available. If standard deviations were not reported the standard deviation was calculated using the following formula: SD = max–min/4 [21]. If the included studies did not include tables describing their outcome measures of central tendency and variations, the first author was contacted, and results were requested. All tests of significance were two-tailed, and an α of less than 0.05 was considered significant. If more than ten studies were included, publication bias was assessed in accordance with the guidelines outlined in the Cochrane Handbook. In such instances, funnel plots, Egger’s regression test and Kendall's tau with continuity correction were subsequently employed [13]. Funnel and forest plots, and all statistical analyses, were performed using STATA SE (Version 13.0; StataCorp, College Station, Texas, USA) for Windows, and the comprehensive meta-analysis software package (CMA), version 3 (Biostat Inc, Englewood, NJ, USA).

## Results

### Study selection and characteristics

The initial literature search identified 1576 studies for consideration. Of those, 621 studies were excluded for duplication, and the titles and abstracts of the remaining 946 publications were checked for eligibility. Five hundred twenty-three (n = 523) studies were excluded after inspection of the titles. These studies were either published study protocols, basic science studies, clinical studies using other non-cultured preparation, or narrative reviews. Of the remaining 423 studies, the abstracts were examined and a further 281 studies were excluded. Of the remaining 142 studies, 39 publications were excluded as they comprised systematic reviews and meta-analyses. Additionally, 19 studies were excluded because they either employed multiple injections, combined various biologics or used stromal vascular fraction (SVF) or micro-fragmented adipose tissue (MFAT) as mesenchymal stromal cells. Furthermore, 74 studies were excluded as they were classified as level IV clinical studies. After all exclusions, ten (n = 10) studies met all the eligibility criteria and were included in the analysis (Supplementary Fig. 1) [9, 22–30]. All ten studies were published in English between 2019 and 2023 with a cumulative total of 563 cases. A total of 281 patients were treated with MSC’s and 282 patients were treated with other biologics. The study characteristics are summarized in Table 1.

**Table 1:** Summary of the Study Characteristics of Level I and II studies

Authors	LOE	Country	MSC	Patients (n)	Control	Patients (n)	Follow-up (months)	Outcomes MFAT-Control
Lamo-Espinoza 2016	II	Spain	BM-MSC 100 ml iliac crest Surface markers for identification: CD 90, CD73, CD 44. Absent Markers: CD34, CD45 20 × 10^6^ 3.0 ml injected	N = 10 F = 6, M = 4 Mean age 65.9	4 ml HA (Hyalone®)	N = 10 F = 3 M = 7 Mean Age 60.3	12	**WOMAC Pain MSC Control** Base 4.5(4–5) 5.5(5–6) 3/12 3(2–5) 3.5(3–7) 6/12 3.5(2–5) 2.5(1–5) 12/12 2.5(2–4) 3.5(3–5) **WOMAC Stiffness MSC Control** Base 2.5(2–4) 2(1–3) 3/12 2(1–2) 2(1–2) 6/12 2(1–3) 0.5(0–2) 12/12 2(1–3) 2(1–2) **WOMAC Function MSC Control** Base 19(12–25) 21(13–24) 3/12 10(7–18) 9(7–11) 6/12 14.5(8–17) 7.5(2–13) 12/12 11(9–14) 9.5(5–23) **WOMAC Total MSC Control** Base 28(16–34) 29(19–38) 3/12 13(11–26) 12(11–14) 6/12 20(13–23) 10(4–20) 12/12 16.5(12–19) 13.5(8–33) **VAS MSC Control** Base 7 (5–8) 5(3–7) 3/12 4 (2–6) 3(2–5) 6/12 3 (1–5) 5 (2–8) 12/12 2 (0–4) 4 (3–5)
Bastos 2019	II	Portugal	BM-MSC iliac crest CFU-F assay + flow cytometry	N = 16 F = 6, M = 10 Mean age 55.7	Corticosteroid	N = 17 F = 8, M = 9 Mean age 55.9	12	**KOOS Pain MSC Control** Base 34.5 + 11.4 40.5 + 19.6 12/12 56.8 + 26.6 59.5 + 22.2 **KOOS Symptoms MSC Control** Base 41.5 + 18.4 47.7 + 17.9 12/12 61.6 + 22.5 56.1 + 22.3 **KOOS ADL MSC Control** Base 31.7 + 19.1 40.7 + 21.0 12/12 58.4 + 27.5 61.6 + 24.4 **KOOS Sports MSC Control** Base 13.0 + 21.0 18.0 + 28.0 12/12 36.6 + 29.5 36.2 + 31.7 **KOOS QOL MSC Control** Base 16.8 + 12.4 16.5 + 16.5 12/12 40.2 + 25.9 32.0 + 29.3 **KOOS Total MSC Control** Base 30.3 + 13.1 36.9 + 17.8 12/12 54.2 + 24.7 54.4 + 22.7
Freitag 2019	I	Australia	60 ml adipose tissue Surface markers for identification: CD 90, CD73, CD 105 3.0 ml single injection 100 × 10^6^ ADMSC	N = 10 F = 6, M = 4 Mean age 54.7	Simple analgesia, weight management, exercise	N = 10 F = 8, M = 2 Mean age 59.1	12	**WOMAC Total MSC Control** Base 59.6 + 17.9 58.8 + 12.8 3//12 82.6 + 11.3 65.7 + 9.1 6/12 83 + 9.9 64.4 + 12.2 12/12 84 + 9.4 59.1 + 12.8 **KOOS Pain MSC Control** Base 53 + 14.5 52.8 + 10.8 3/12 77.4 + 15.8 54.9 + 7.4 6/12 76.4 + 12.4 55.3 + 11.4 12/12 77.3 + 11.3 48.9 + 12.7 **KOOS Symptoms MSC Control** Base 63.6 + 21.3 46.1 + 11 3/12 79.6 + 12.9 48.1 + 13.1 6/12 80.1 + 13.7 45.3 + 13 12/12 84.3 + 9.4 47.9 + 13.6 **KOOS ADL MSC Control** Base 58.8 + 19.8 59.4 + 13.6 3/12 82.5 + 12.3 67.1 + 9.8 6/12 83.6 + 9.6 65.5 + 14.4 12/12 84.3 + 9.4 60.7 + 13.5 **KOOS Sports MSC Control** Base 39 + 26.2 26 + 20.4 3/12 63.8 + 22.5 27.5 + 21.9 6/12 66.9 + 15.3 31 + 29.8 12/12 70 + 17.8 31.5 + 33 **KOOS QOL MSC Control** Base 29.4 + 20.5 30.1 + 15.9 3/12 51.6 + 23.7 29.9 + 14.6 6/12 63.3 + 12.2 31.9 + 19.7 12/12 61.8 + 13 33.9 + 18.9 **VAS MSC Control** Base 6.5 6.5 3/12 2.6 6 6/12 2.8 6 12/12 2.7 6
Lee 2019	I	Poland	20 ml adipose tissue Surface markers for identification: CD 31,CD34, CD45, CD 73, CD90 1 × 10^8^ 3.0 ml injected	N = 12 F = 9, M = 3 Mean age 62.2	3 ml Saline	N = 12 F = 9, M = 3 Mean age 63.2	6	**WOMAC Total MSC Control** Base 60 + 17.0 56 3/12 39 57 6/12 26.7 + 13.3 52 **WOMAC Pain MSC Control** Base 15 16 3/12 6 11 6/12 5 10 **WOMAC Stiffness MSC Control** Base 6 6 3/12 4 5 6/12 2 4 **WOMAC Function MSC Control** Base 40 47 3/12 29 37 6/12 33 20 **VAS MSC Control** Base 7 6 3/12 5 6 6/12 4 6 **KOOS Pain MSC Control** Base 5 5 3/12 6 5 6/12 8 6 **KOOS Symptoms MSC Control** Base 46 46 3/12 63 47 6/12 70 49 **KOOS ADL MSC Control** Base 50 54 3/12 63 58 6/12 71 61 **KOOS Sports MSC Control** Base 19 28 3/12 34 27 6/12 48 30 **KOOS QOL MSC Control** Base 25 32 3/12 45 45 6/12 58 40 **VAS MSC Control** Base 6.8 5.4 3/12 4.5 5.2 6/12 3.4 5.2
Lu 2019	II	China	Re-Join® Liposuction Characterized according to ISCT	N = 26 F = 23, M = 3 Mean age 55.0	HA: ARTZ 25 mg/25 ml	N = 26 F = 23, M = 3 Mean age 59.6	12	**WOMAC Total MSC Control** Base 30.83 + 19.14 34.17 + 17.16 6/12 21.7 + 17.87 27.58 + 16.93 12/12 21.35 + 18.19 27.25 + 16.33 **WOMAC Pain MSC Control** Base 7.7 + 4.1 7.2 + 3.7 6/12 5.1 + 3.1 5.9 + 3.6 12/12 4.7 + 3.4 5.9 + 3.4 **WOMAC Stiffness MSC Control** Base 2.4 + 1.9 2.6 + 1.8 6/12 1.7 + 2.1 2.1 + 1.8 12/12 1.6 + 2.2 2.2 + 1.8 **WOMAC Function MSC Control** Base 23.6 + 14.6 22.3 + 13.3 6/12 17.0 + 13.4 18.5 + 12.8 12/12 15.7 + 13.4 18.2 + 12.2 **VAS MSC Control** Base 5.4 + 2.3 4.9 + 2.5 6/12 2.8 + 2.6 4.2 + 2.6 12/12 2.8 + 2.6 4.4 + 2.4
Yokota 2019 Koos 4	III	Japan	Adipose Tissue-Derived Cultured Stem Cells 100 ml liposuction 12.8 mill cells for knee injection	N = 42 F = 33, M = 9 Mean age 70	Stromal Vascular Fraction	N = 38 F = 31, M = 7 Mean age 73	6	**KOOS Pain MSC Control** Base 50 42 3/12 62 60 6/12 60 62 **KOOS Symptoms MSC Control** Base 54 45 3/12 60 58 6/12 67 64 **KOOS ADL MSC Control** Base 60 60 3/12 68 68 6/12 68 74 **KOOS Sports MSC Control** Base 18 20 3/12 30 24 6/12 24 34 **KOOS QOL MSC Control** Base 25 19 3/12 43 43 6/12 40 30 **VAS MSC Control** Base 7 8 3/12 4 5 6/12 3 4
Lamo Espinoza 2020	II	Spain	BM-MSC iliac crest Surface markers for identification: CD 90, CD73, CD 44 Absent Markers: CD34, CD45 Flow cytometry	N = 24 F = 8, M = 16 Mean age 54	PRP PRGF-Endoret	N = 26 F = 9, M = 17 Mean age 56	12	**VAS MSC Control** Base 5.3 + 1.9 5 + 1.8 3/12 3.8 + 2 3.8 + 1.6 6/12 3.3 + 2.2 3.5 + 2 12/12 3.5 + 2.5 4.5 + 2.2 **WOMAC Total MSC Control** Base 33.4 + 18.7 31.9 + 16.2 3/12 24.4 + 17.4 21.7 + 17.1 6/12 21.3 + 16.6 23 + 15 12/12 16.7 + 11.6 22.3 + 15.8 **WOMAC Pain MSC Control** Base 7.7 + 4.1 7.2 + 3.7 6/12 5.1 + 3.1 5.9 + 3.6 12/12 4.7 + 3.4 5.9 + 3.4 **WOMAC Stiffness MSC Control** Base 3.3 + 2.1 3 + 1.6 3/12 2.3 + 2.2 1.9 + 1.4 6/12 2 + 1.9 2.2 + 1.6 12/12 2.1 + 1.9 2.1 + 1.6 **WOMAC Function MSC Control** Base 23.5 + 13.2 22.3 + 12.8 3/12 17.6 + 12.6 15.5 + 12.6 6/12 14.9 + 11.8 16.3 + 11.1 12/12 23 + 16.6 22.3 + 15.8
Ho 2022	II	Hongkong	BM-MSC Histology	N = 10 F = 4, M = 6 Mean age 56.7	HA 6 ml Synvisc	N = 10 F = 8, M = 16 Mean age 54	12	**VAS MSC Control** Base 2.5 + 0.7 1.5 + 0.6 3/12 2.2 + 1.6 3.1 + 2.0 6/12 2.0 + 3.0 3.9 + 2.0 12/12 2.0 + 3.8 4.2 + 2.1 **WOMAC Total MSC Control** Base 40.7 + 26 38.1 + 20.2 3/12 38.6 + 15.5 29.6 + 14.8 6/12 33.6 + 24.2 37.7 + 22.2 12/12 30.3 + 17.4 29.8 + 20.3 **WOMAC Pain MSC Control** Base 8.2 + 5.4 7.6 + 3 3/12 8.0 + 4.0 6.0 + 2.2 6/12 7.1 + 5.7 7.2 + 3.2 12/12 6.4 + 4.5 4.4 + 3.2 **WOMAC Stiffness MSC Control** Base 3.8 + 2.5 4.2 + 1.9 3/12 3.7 + 1.5 3.2 + 2.4 6/12 3.1 + 2.1 2.8 + 2.9 12/12 3.3 + 1.6 3.9 + 2.5 **WOMAC Function MSC Control** Base 28.7 + 18.7 32.9 + 15.17 3/12 27.0 + 10.9 27.0 + 13.2 6/12 23.4 + 16.9 24.9 + 25.8 12/12 20.6 + 12.2 30.3 + 18.3
Schweich 2022	II	Brasil	adipose tissue Flow Cytometry Pro: CD105, CD90, CD34, CD133	N = 6 F = 2, M = 4 Mean age 48.0	PRP 18 ml blood 1500 rpm: 10 min 2000 rpm:15 min	N = 6 F = 2, M = 4 Mean age 58.5	6	**VAS MSC Control** Base 2.5 + 0.7 1.5 + 0.6 6/12 2.0 + 3.0 3.9 + 2.0
Kim 2023	I	Korea	Adipose tissue CD312, CD 34, CD45 CD73, CD90 3 ml 1 × 10^8^ in 3 ml saline	N = 125 F = 39, = 86 Mean age 63.7	Normal Saline 3 ml	N = 127 F = 26,M = 101 Mean age 63.8	6	**WOMAC Total MSC Control** Base 55.0 + 15.9 51.7 + 15.9 3/12 35.1 + 18.7 38.0 + 17.2 6/12 33.3 + 18.6 37.4 + 19.2 **WOMAC Pain MSC Control** Base 10.7 + 3.3 11.3 + 3.2 3/12 8.7 + 4.1 8.6 + 3.8 6/12 6.4 + 4.0 8.6 + 4.4 **WOMAC Stiffness MSC Control** Base 4.5 + 1.3 4.9 + 1.5 3/12 3.1 + 1.8 3.6 + 1.6 6/12 2.7 + 1.9 3.6 + 1.9 **WOMAC Function MSC Control** Base 39.8 + 9.4 41.8 + 10.3 3/12 26.5 + 18.7 32.1 + 12.1 6/12 18.1 + 18.6 27.5 + 19.2 **VAS MSC Control** Base 57.7 + 17.1 60.9 + 16.6 3/12 35.5 + 24.6 47.7 + 23.7 6/12 32.5 + 24.6 46.6 + 19.2 **KOOS Pain MSC Control** Base 50.1 + 13.9 46.9 + 16.2 3/12 34.8 + 18.2 35.8 + 17.2 6/12 33.8 + 17.0 36.9 + 17.1 **KOOS Symptoms MSC Control** Base 55.7 + 15.9 51.7 + 15.9 3/12 44.7 + 18.3 42.9 + 17.0 6/12 40.7 + 17.9 41.9 + 17.5 **KOOS ADL MSC Control** Base 53.7 + 14.8 50.2 + 17.0 3/12 38.0 + 18.6 39.8 + 16.5 6/12 40 + 17.3 39.2 + 17.0 **KOOS Sports MSC Control** Base 23.6 + 18.3 21.5 + 19.0 3/12 10.1 + 21.7 16.9 + 21.6 6/12 7.6 + 22.4 15.6 + 20.0 **KOOS QOL MSC Control** Base 32.9 + 14.3 31.8 + 16.1 3/12 20.3 + 16.4 5.9 + 14.9 6/12 19.5 + 17.3 23.3 + 15.6

50.1

#### Risk of bias cochrane assessment tool version 2

Two studies were assessed as having a high risk of bias due to confounding [29, 30]. One study had some risk of bias [24] due to confounding, missing data and bias in the measurements of outcome. The remaining seven studies had a low risk of bias (Table 2) [19, 22–28].

**Table 2:** Risk of bias robins cochrane risk of bias assessment tool version 2

Authors	LOE	Bias due to confounding	Bias in Selection of Participants	Bias in Classification of interventions	Bias due to deviations from Intended Interventions	Bias due to missing data	Bias in Measurement of outcomes	Bias in Selection of the Reported Results	Overall bias
Lamo Espinoza	II	Low	Low	Low	Low	Low	Low	Low	Low
Bastos 2019	II	Low	Low	Low	Moderate	Low	Low	Low	Low
Freitag 2019	II	Low	Low	Low	Moderate	Low	Low	Low	Low
Lee 2019	II	Low	Low	Low	Low	Low	Low	Low	Low
Lu 2019	II	Low	Low	Low	Low	Low	Low	Low	Low
Yokota 2019	III	High	Some	Some	Low	Low	Low	Low	High
Lamo Espinoza 2020	II	Low	Low	Low	Low	Low	Low	Low	Low
Ho 2022	II	Some	Low	Low	Low	Some	Some	Low	Some
Schweich 2022	II	High	Low	Low	Moderate	Moderate	Moderate	Low	High
Kim 2023	II	Low	Low	Low	Low	Low	Low	Low	Low

#### Publication bias

The funnel plot exhibited asymmetry in the standard deviations of the means, suggesting the presence of publication bias (Supplementary Fig. 2). This finding is corroborated by Egger's regression test, which yielded an intercept of 3.26447 with a t-value of 3.75116 and a *p *value of 0.00716. Additionally, Kendall's tau with continuity correction showed a tau coefficient of 0.58 with a *p* value of 0.02857, further supporting the indication of publication bias.

#### Quality assessment

The Grade quality assessment is summarized in Table 3. Nine included studies were initially categorized as high level of certainty [9, 22–29] and one study was categorized as low level of certainty [30]. The study by Yokota [30] was a Level 3 comparative observational study with no randomization. Due to the high risk of bias, two studies [29, 30] were downgraded to a very low level of certainty. Three studies [22–24] were downgraded to a moderate level of certainty due to some inconsistency and imprecision of results. The remaining five studies maintained their initial high level of certainty in the assessment [9, 25–28]. Given the overall high and moderate final level of certainty, the confidence in the effect estimate is therefore moderate to high and it is likely that the true effect may not be substantially different from the estimate of the effect.

**Table 3:** Quality assessment using the cochrane GRADE system

Authors	LOE	Initial level of certainty	Final level of certainty	Risk of bias	Inconsistency of results	Indirectness of evidence	Imprecision of Results	Large Effects (Upgrading)	Dose Response (Upgrading)	Opposing Plausible residual Bias and confounding (upgrading)
Lamo Espinoza	II	High	High	Low	Low	Low	Low	–	–	Low
Bastos 2019	II	High	Moderate	Low	Some	Low	Low	–	–	Low
Freitag 2019	II	High	Moderate	Low	Some	Low	Low	–	–	Low
Lee 2019	II	High	High	Low	Low	Low	Low	–	–	Low
Lu 2019	II	High	High	Low	Low	Low	Low	–	–	Low
Yokota 2019	III	Low	Very Low	High	Low	Some	Low	–	–	Low
Lamo Espinoza 2020	II	High	High	Low	Low	Low	Low	–	–	Low
Ho 2022	II	High	Moderate	Some	Low	Low	Some	–	–	Low
Schweich 2022	II	High	Very Low	High	Some	Low	Some	–	–	Low
Kim 2023	II	High	High	Low	Low	Low	Low	–	–	Low

Quality assessment using the CMS score (Table 4) revealed that only three out of the ten included studies were of high quality [9, 22, 24]. The primary limitations of these studies were the short mean follow-up period, small sample size, and inadequate description of the post-intervention rehabilitation protocol. Five studies [23, 25–28] were assessed as having good study quality. Similar to the low-quality studies, the main limitations included a short mean follow-up period, small sample size, and inadequate description of the post-intervention rehabilitation protocol. Only two studies [29, 30] were assessed as having fair quality, with limitations also related to the previously mentioned issues.

**Table 4:** Modified Coleman methodology score

Authors	Total points	Study size	Mean follow-Up	Percent of patients with follow-up	Number of interventions	Type of study	Diagnostic certainty	Description surgical technique	Description post op rehabilitation	Outcome criteria	Procedures for assessing outcomes	Description of subject selection
Lamo Espinoza 2016	73	0	0	5	10	15	5	5	0	7	11	15
Bastos 2019	94	4	0	5	10	15	5	5	10	10	15	15
Freitag 2019	75	4	0	5	10	15	5	5	3	7	11	10
Lee 2019	72	4	0	5	10	15	5	5	0	7	11	10
Lu 2019	75	4	0	5	10	15	5	3	0	7	11	15
Yokota 2019	67	10	0	5	10	0	5	3	3	10	6	15
Lamo Espinoza 2020	77	4	0	5	10	15	5	5	0	7	11	15
Ho 2022	84	4	0	5	10	15	5	5	0	10	15	15
Schweich 2022	67	4	0	5	10	15	5	5	0	7	11	5
Kim 2023	85	10	0	5	10	15	5	5	0	10	15	10
Overall Mean	77											

### Outcome scores: VAS

The clinical outcomes for VAS for all studies are summarized in Table 1. Nine studies reported on baseline figures and the pooled estimates did not demonstrate significant between group differences (SMD 0.395, 95% CI − 0.033 to 0.823, *p* = 0.07, *I*^2^ = 76%; Supplementary Fig. 3) [22–30].

**Fig. 1:**
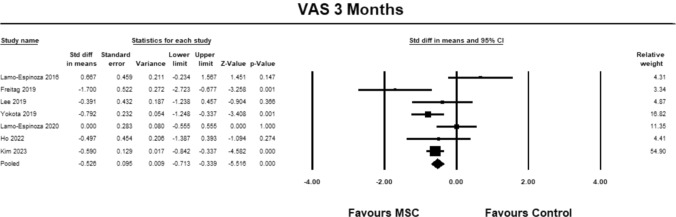
Forest Plot comparing VAS at 3 months demonstrated between group significant differences (*p* = 0.0001) in favor of MSCs

Seven studies were included to analyse VAS at 3 months [9, 23–27, 30]. The pooled estimate demonstrated significant between group differences in favour of MSC (SMD − 0.526; 95% CI − 0.713 to − 0.339, *p* = 0.0001, *I*^2^ = 64%; Fig. 1).

**Fig. 2:**
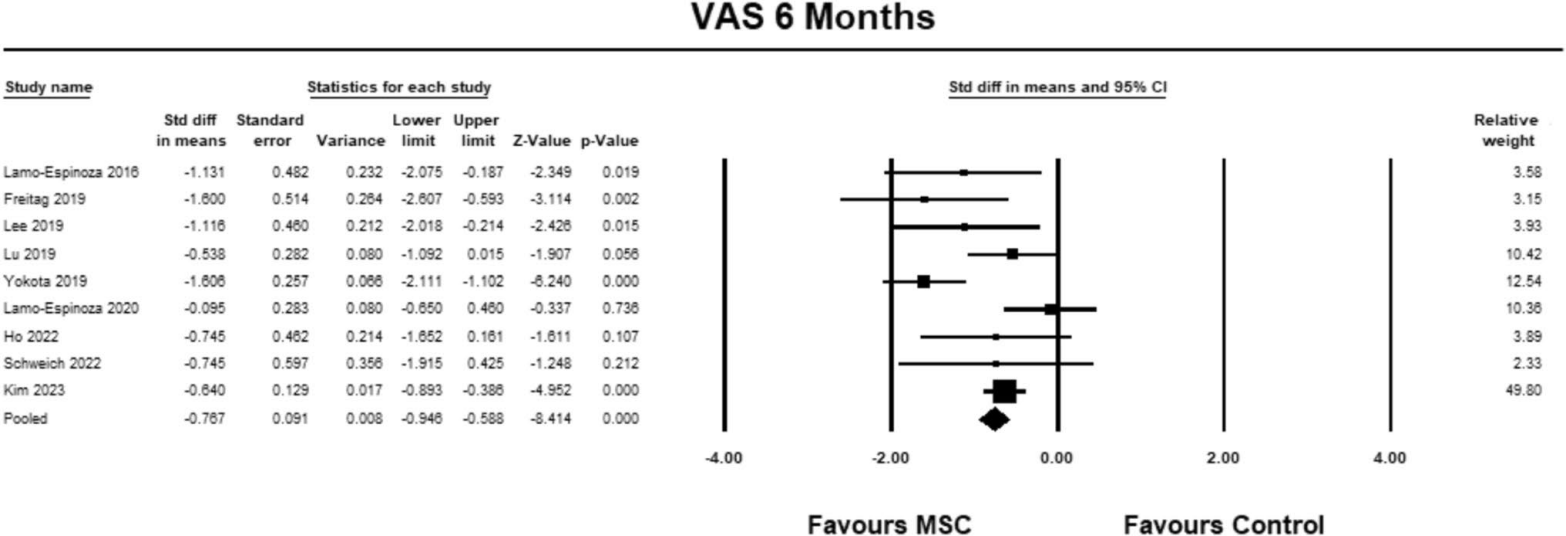
Forest Plot comparing VAS at 6 months demonstrated between group significant differences (*p* = 0.0001) in favor of MSCs

Nine studies were included to analyze VAS at 6 months [9, 23–30] The pooled estimate demonstrated significant between group differences in favour of MSC (SMD − 0.767; 95% CI − 0.946 to − 0.588, *p* = 0.0001, *I*^2^ = 63%; Fig. 2).

Five studies were included to analyze VAS at 12 months [23–26, 28]. The pooled estimate demonstrated significant between group differences in favour of MSC (SMD − 0.952; 95% CI − 1.491 to − 0.413; *p* = 0.001, *I*^2^ = 58%; Supplementary Fig. 4).

### Outcome scores: KOOS

#### KOOS pain

The clinical outcomes for the KOOS sub-scores for all studies are summarized in Table 1. For the KOOS pain sub-score, five studies [9, 22, 23, 27, 30] were included for analysis at baseline. The pooled estimate demonstrated significant differences in favor of the control group (SMD 0.230, 95% CI 0.033 to 0.426, *p* = 0.02, *I*^2^ = 5%; Supplementary Fig. 5). Although the SMD favored MSC at baseline, the magnitude of effect was small, suggesting that the differences were not relevant [13].

**Fig. 3:**
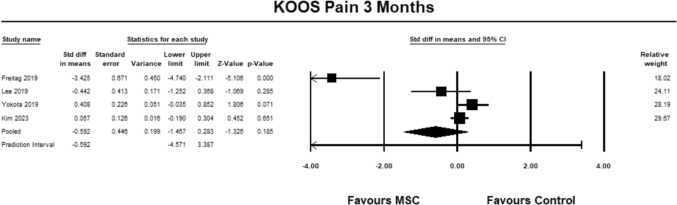
Forest Plot comparing the KOOS subscale pain at 3 months demonstrated non-significant between group differences in favor of the control group (*p* = 0.185)

For the KOOS pain sub-score, four studies [9, 23, 27, 30] were included for analysis at 3 months. The pooled estimate demonstrated non-significant between group differences but favored MSC (SMD − 0.592, 95% CI − 1.467 to 0.283 *p* = 0.185, *I*^2^ = 42%; Fig. 3). Although the SMD favored MSC at 3 months baseline, the magnitude of effect was small, suggesting that the differences were not relevant [13].

**Fig. 4:**
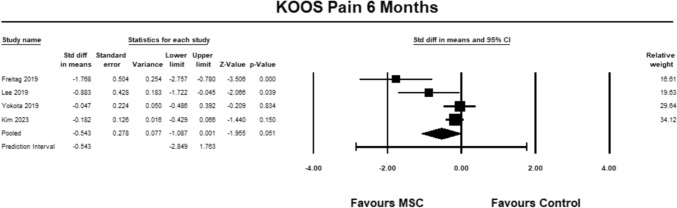
Forest Plot comparing the KOOS subscale pain at 6 months demonstrated near significant between group differences in favor of MSCs (*p* = 0.051)

For the KOOS pain sub-score, four studies [9, 23, 27, 30] were included for analysis at 6 months. The pooled estimate demonstrated near significant between group differences in favor of MSC (SMD − 0.543, 95% CI − 1.087 to 0.001, *p* = 0.051, *I*^2^ = 42%; Fig. 4). The magnitude of effect was moderate, suggesting that the differences were relevant [13].

#### KOOS symptoms

For the KOOS symptoms sub-score, five studies [9, 22, 23, 27, 30] were included for analysis at baseline. The pooled estimate did not demonstrate significant between group differences (SMD − 0.330, 95% CI − 0.685 to 0.026, *p* = 0.069, *I*^2^ = 61%; Supplementary Fig. 6).

**Fig. 5:**
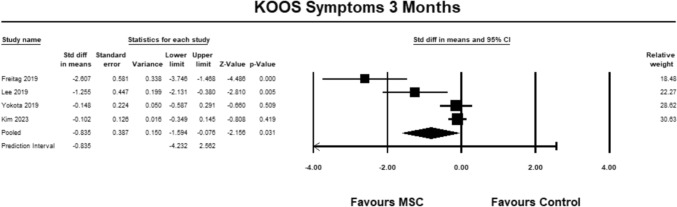
Forest Plot comparing the KOOS subscale symptoms at 3 months demonstrated significant between group differences in favor of MSCs (*p* = 0.031)

For the KOOS symptoms sub-score, four studies [9, 23, 27, 30] were included for analysis at 3 months. The pooled estimate demonstrated significant between group differences in favor of MSC (SMD − 0.835, 95% CI − 1.594 to − 0.076, *p* = 0.031, *I*^2^ = 512%; Fig. 5). The magnitude of effect was large, suggesting that the differences were relevant.

**Fig. 6:**
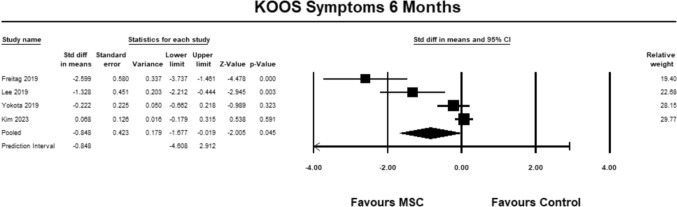
Forest Plot comparing the KOOS subscale symptoms at 6 months demonstrated significant between group differences in favor of MSCs (*p* = 0.045)

For the KOOS symptoms sub-score, four studies [9, 23, 27, 30] were included for analysis at 6 months. The pooled estimate demonstrated significant between group differences in favor of MSC (SMD − 0.848, 95% CI − 1.677 to − 0.019, *p* = 0.045, *I*^2^ = 47%; Fig. 6). The magnitude of effect was large, suggesting that the differences were relevant.

#### KOOS ADL

For the KOOS ADL sub-score, five studies [9, 22, 23, 27, 30] were included for analysis at baseline. The pooled estimate did not demonstrate significant between group differences (SMD − 0.122, 95% CI − 0.487 to 0.243, *p* = 0.511, *I*^2^ = 56%; Supplementary Fig. 7).

**Fig. 7:**
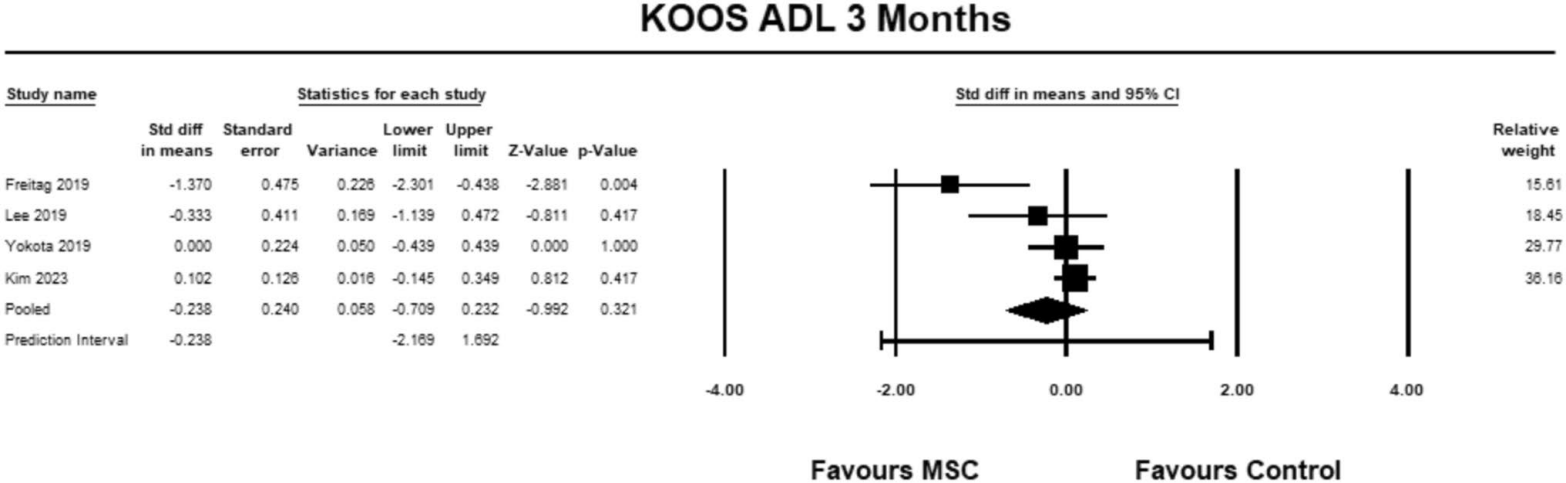
Forest Plot comparing the KOOS subscale ADL at 3 months could not demonstrate significant between group differences (*p* = 0.321)

For the KOOS ADL sub-score, four studies [9, 23, 27, 30] were included for analysis at 3 months. The pooled estimate did not demonstrate significant between group differences (SMD − 0.238, 95% CI − 0.709 to 0.232, *p* = 0.321, *I*^2^ = 68%; (Fig. 7).

**Fig. 8:**
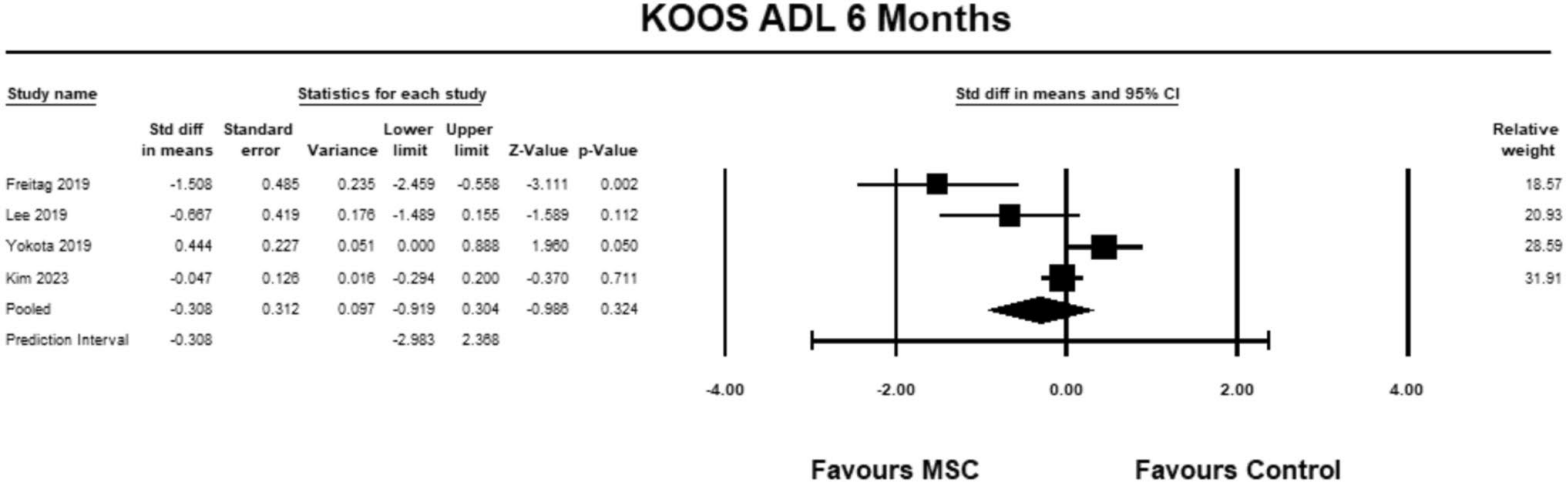
Forest Plot comparing the KOOS subscale ADL at 6 months could not demonstrate significant between group differences (*p* = 0.324)

For the KOOS ADL sub-score, four studies [9, 23, 27, 30] were included for analysis at 6 months. The pooled estimate did not demonstrate significant between group differences (SMD − 0.308, 95% CI − 0.919 to 0.304, *p* = 0.324, *I*^2^ = 81%; Fig. 8).

#### KOOS sports

For the KOOS sub-score sports, five studies [9, 22, 23, 27, 30] were included for analysis at baseline. The pooled estimate did not demonstrate significant between group differences (SMD 0.278, 95% CI − 0.245 to 0.800, *p* = 0.298, *I*^2^ = 78%; Supplementary Fig. 8).

**Fig. 9:**
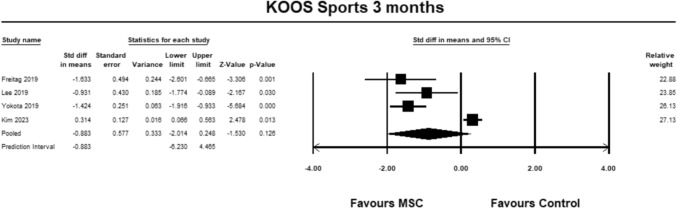
Forest Plot comparing the KOOS subscale sports at 3 months could not demonstrate significant between group differences (*p* = 0.126)

For the KOOS sub-score sports, four studies [9, 23, 27, 30] were included for analysis at 3 months. The pooled estimate did not demonstrate significant between group differences (SMD − 0.883, 95% CI − 2.014 to 0.248, *p* = 0.126, *I*^2^ = 94%; Fig. 9).

**Fig. 10:**
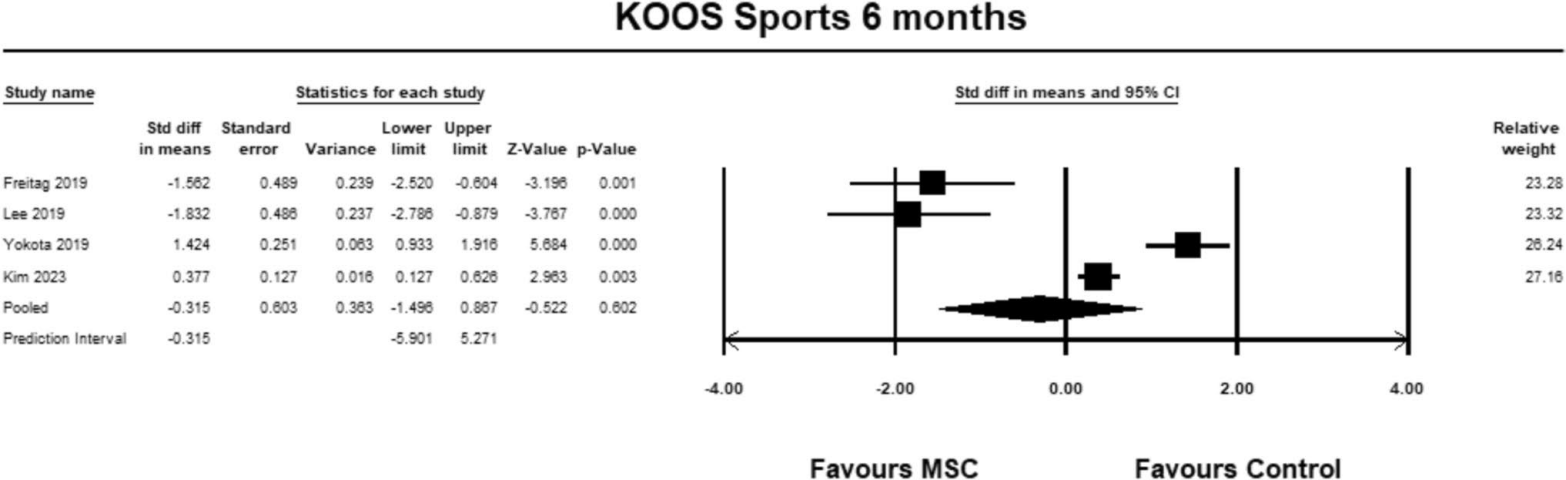
Forest Plot comparing the KOOS subscale sports at 6 months could not demonstrate significant between group differences (*p* = 0.602)

For the KOOS sub-score sports, four studies [9, 23, 27, 30] were included for analysis at 6 months. The pooled estimate did not demonstrate significant between group differences (SMD − 0.315, 95% CI − 1.496 to 0.867, *p* = 0.602, *I*^2^ = 94%; (Fig. 10).

#### KOOS quality of life

For the KOOS QOL sub-score, five studies [9, 22, 23, 27, 30] were included for analysis at baseline. The pooled estimate did not demonstrate significant between group differences (SMD − 0.182, 95% CI − 0.506 to 0.142, *p* = 0.272, *I*^2^ = 46%; Supplementary Fig. 9).

**Fig. 11:**
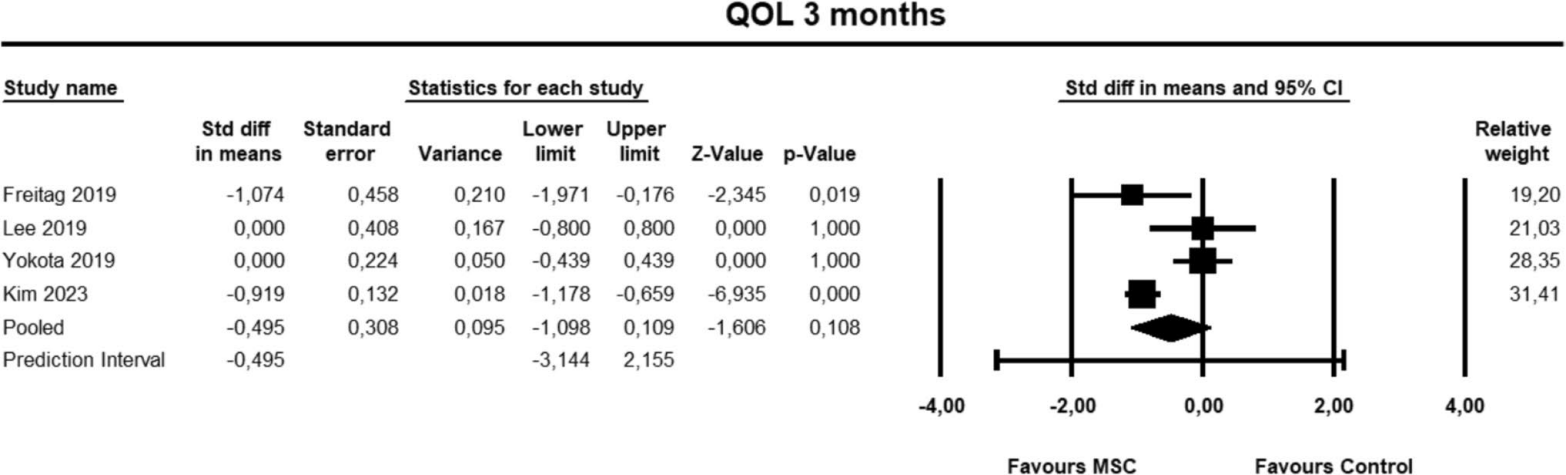
Forest Plot comparing the KOOS subscale QOL at 3 months could not demonstrate significant between group differences (*p* = 0.108)

For the KOOS QOL sub-score at 3 months, four studies [9, 23, 27, 30] were included for analysis at 3 months. The pooled estimate did not demonstrate significant between group differences (SMD − 0.495, 95% CI − 1.098 to 0.109, *p* = 0.108, *I*^2^ = 81%; Fig. 11).

**Fig. 12:**
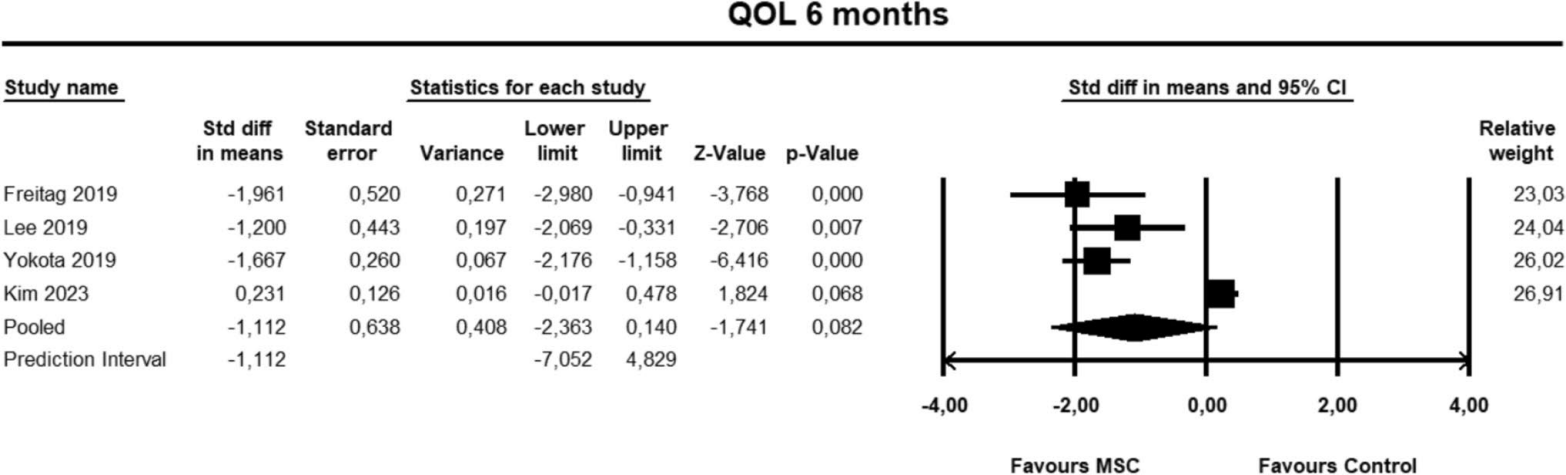
Forest Plot comparing the KOOS subscale QOL at 12 months could not demonstrate significant between group differences (*p* = 0.082)

For the KOOS QOL sub-score, four studies [9, 23, 27, 30] were included for analysis at 6 months. The pooled estimate did not demonstrate significant between group differences (SMD − 1.112, 95% CI − 2.363 to 0.140, *p* = 0.082, *I*^2^ = 95%; (Fig. 12).

### Outcome scores: WOMAC

#### WOMAC total

For the WOMAC total score, seven studies [9, 23–28] were included for analysis at baseline. The pooled estimate did not demonstrate significant between group differences (SMD 0.119, 95% CI − 0.069 to 0.307, *p* = 0.215, *I*^2^ = 0%; Supplementary Fig. 10).

**Fig. 13:**
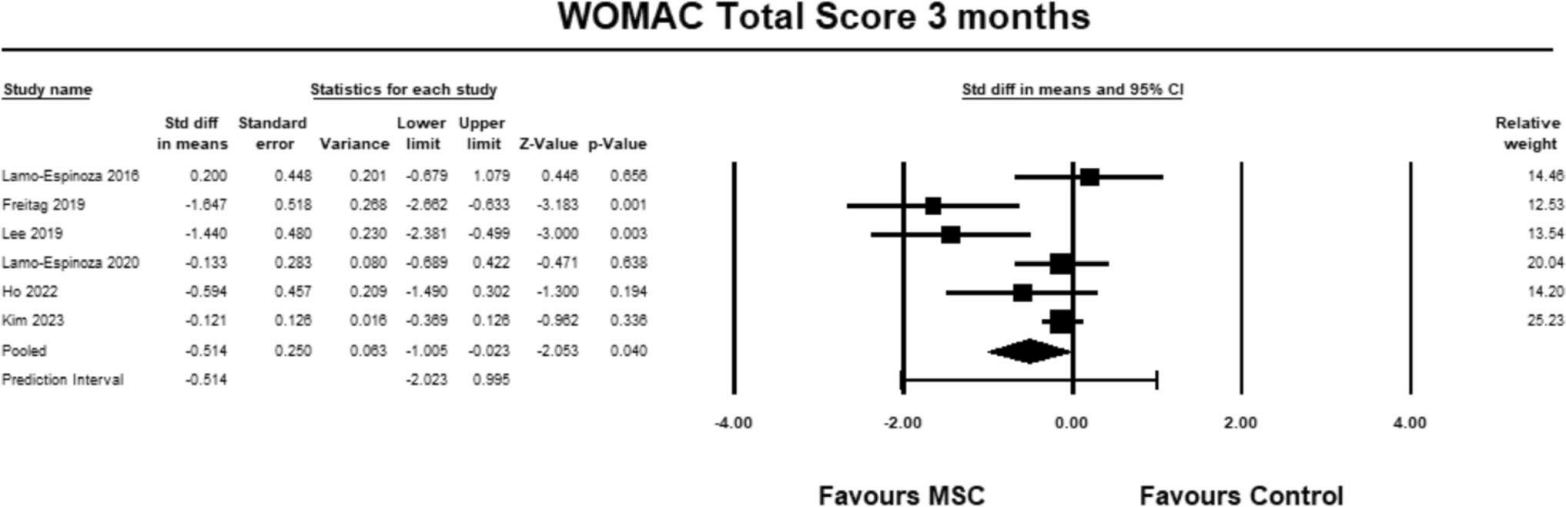
Forest Plot comparing the total WOMAC score at 3 months demonstrated significant between group differences in favor of MSCs (*p* = 0.04)

For the WOMAC total score, six studies [9, 23–28] were included for analysis at 3 months. The pooled estimate demonstrated significant between group differences in favour of MSC. (SMD − 0.514, 95% CI − 1.005 to − 0.023, *p* = 0.040, *I*^2^ = 78%; Fig. 13). The magnitude of effect was medium, suggesting that the differences were relevant.

**Fig. 14:**
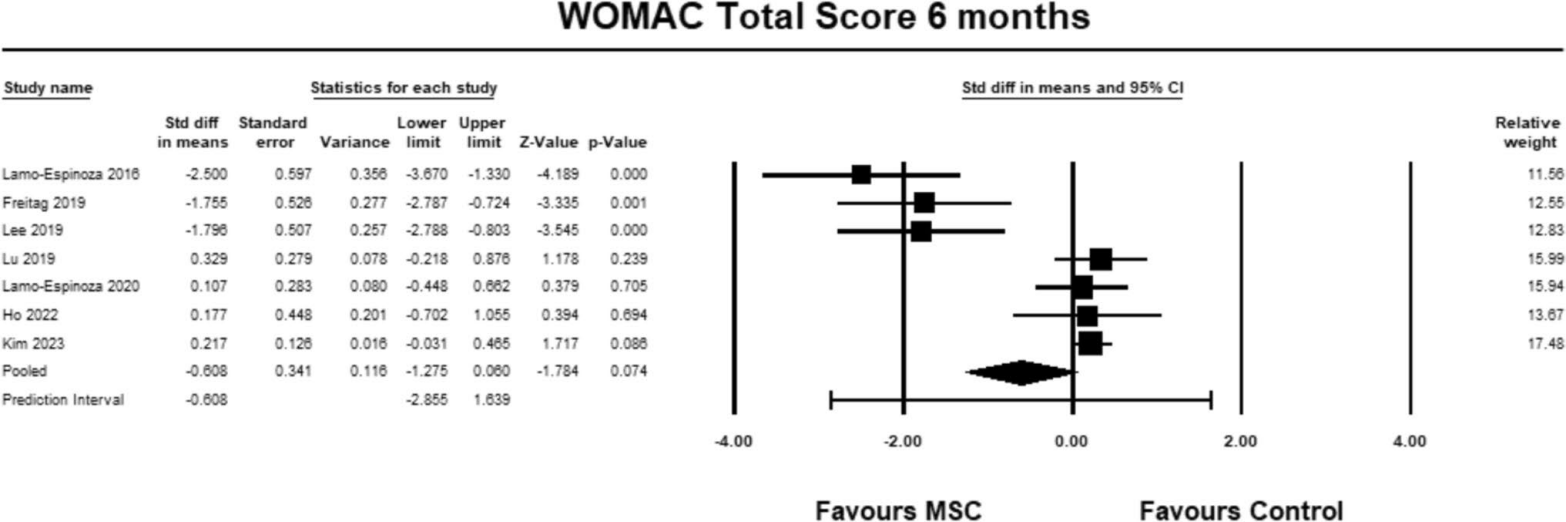
Forest Plot comparing the total WOMAC score at 6 months demonstrated near significant between group differences in favor of MSCs (*p* = 0.074)

For the WOMAC total score, seven studies [9, 23–28] were included for analysis at 6 months. The pooled estimate reached near significance between groups in favour of MSC. (SMD − 0.608, 95% CI − 1.275 to 0.060, *p* = 0.074, *I*^2^ = 96%; Fig. 14). The magnitude of effect was medium, suggesting that the differences were relevant.

**Fig. 15:**
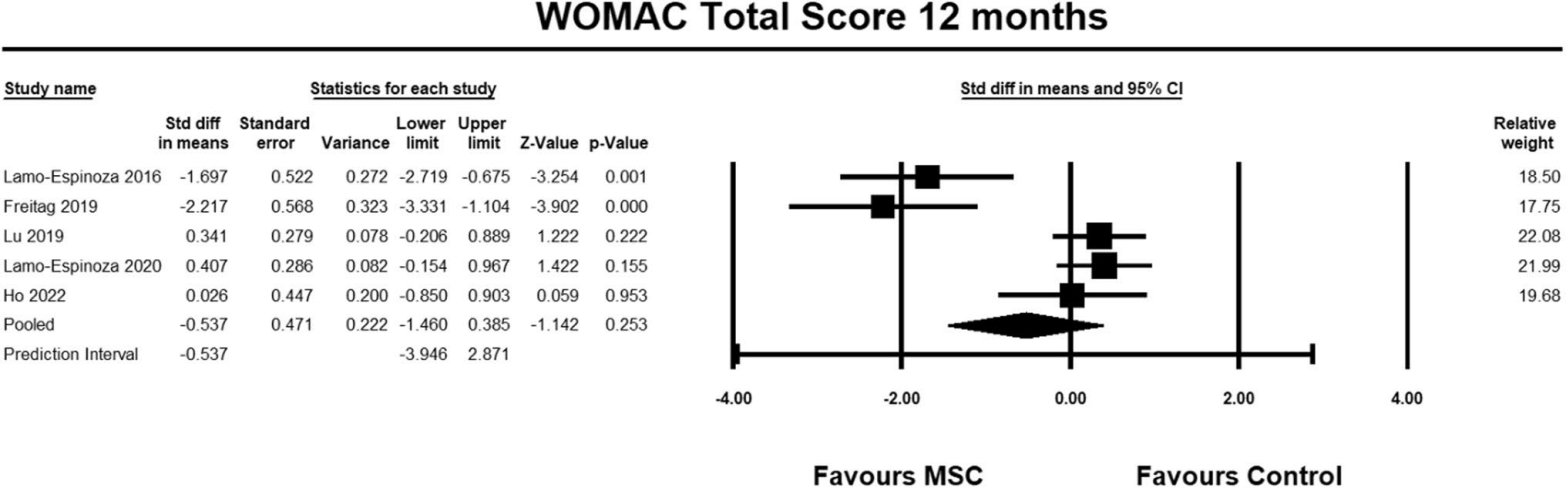
Forest Plot comparing the total WOMAC score at 12 months did not demonstrate significant between group differences (*p* = 0.253)

For the WOMAC total score, five studies [23–27] were included for analysis at 12 months. The pooled estimate did not demonstrate between group differences (SMD − 0.537, 95% CI − 1.460 to 0.385, *p* = 0.253, *I*^2^ = 86%; Fig. 15).

#### WOMAC pain

For the WOMAC pain score, six studies [9, 24–28] were included for analysis at baseline. The pooled estimate did not demonstrate significant between group differences (SMD − 0.430, 95% CI − 1.043 to 0.183, *p* = 0.169, *I*^2^ = 84%; Supplementary Fig. 11).

For the WOMAC pain score, four studies [9, 24, 25, 27] were included for analysis at 3 months. The pooled estimate did not demonstrate significant between group differences (SMD − 0.314, 95% CI − 1.124 to 0.496, *p* = 0.448, *I*^2^ = 49%; Supplementary Fig. 12).

For the WOMAC pain score, six studies [9, 24–28] were included for analysis at 6 months. The pooled estimate did not demonstrate significant between group differences (SMD − 0.443, 95% CI − 1.065 to 0.178 *p* = 0.162, *I*^2^ = 80%; Supplementary 13).

For the WOMAC pain score, four studies [24–27] were included for analysis at 12 months. The pooled estimate did not demonstrate significant between group differences (SMD − 0.305, 95% CI − 0.776 to 0.165 *p* = 0.204, *I*^2^ = 49%; Supplementary Fig. 14).

#### WOMAC stiffness

For the WOMAC stiffness score, six studies [9, 24–28] were included for analysis at baseline. The pooled estimate did not demonstrate significant between group differences (SMD − 0.155, 95% CI − 0.5333 to 0.040, *p* = 0.119, *I*^2^ = 0%; Supplementary Fig. 15).

For the WOMAC stiffness score, five studies [9, 24–27] were included for analysis at 3 months. The pooled estimate reached near significance in favour of MSC (SMD − 0.192, 95% CI − 0.402 to 0.017 *p* = 0.072, *I*^2^ = 0%; Supplementary Fig. 16).

For the WOMAC stiffness score, six studies [9, 24–28] were included for analysis at 6 months. The pooled estimate did not demonstrate significant between group differences (SMD − 0.104, 95% CI − 0.596 to 0.388, *p* = 0.678, *I*^2^ = 0%; Supplementary Fig. 17).

For the WOMAC stiffness score, four studies [24–26, 28] were included for analysis at 12 months. The pooled estimate did not demonstrate significant between group differences (SMD 0.067, 95% CI − 0.655 to 0.789, *p* = 0.856, *I*^2^ = 32%; Supplementary Fig. 18).

#### WOMAC function

For the WOMAC function score, six studies [9, 24–28] were included for analysis at baseline. The pooled estimate did not demonstrate significant between group differences (SMD − 0.173, 95% CI − 0.369 to 0.022 *p* = 0.082, *I*^2^ = 15%; Supplementary Fig. 19).

For the WOMAC function score, six studies [9, 24–28] were included for analysis at 3 months. The pooled estimate did not demonstrate significant between group differences (SMD 0.012, 95% CI − 0.341 to 0.365 *p* = 0.946, *I*^2^ = 50%; Supplementary Fig. 20).

For the WOMAC function score, six studies [9, 24–28] were included for analysis at 6 months. The pooled estimate did not demonstrate significant between group differences (SMD − 0.632, 95% CI − 1.411 to 0.146 *p* = 0.112, *I*^2^ = 89%; Supplementary Fig. 21).

For the WOMAC function score, four studies [24–27] were included for analysis at 12 months. The pooled estimate did not demonstrate significant between group differences (SMD 0.117, 95% CI − 0.214 to 0.448; *p* = 0.488, *I*^2^ = 0%; Supplementary Fig. 22).

## Discussion

The results of this meta-analysis demonstrated a significant effect of adipose and bone marrow-derived stromal cell (MSC) injections on pain reduction, as measured by the Visual Analog Scale (VAS) for pain, at all assessed time points. Furthermore, MSC injections showed superiority over other non-surgical treatment options. However, these significant differences were not observed in the pain subscales of the Knee injury and Osteoarthritis Outcome Score (KOOS) and the Western Ontario and McMaster Universities Osteoarthritis Index (WOMAC). Only the KOOS pain subscale corroborated the findings of the VAS pain score, demonstrating significantly reduced symptoms in the MSC group. In addition, the lower pain scores were not associated with any clinical differences. This conclusion is based on the analysis of KOOS subscores (ADL, Sport, and QoL) and WOMAC subscores (Stiffness and Function), which reflect functional outcomes. None of the comparisons demonstrated statistically significant differences, suggesting that MSC injection therapy does not result in functional improvement.

Quality assessment of the included studies, using both the GRADE approach and CMS score, indicated that 80% were of high or moderate quality. Additionally, 70% of the studies were assessed as having a low risk of bias, supporting the conclusion that the overall risk of bias across the included studies was low. According to the Cochrane Handbook, the confidence in the effect estimate can be classified as moderate to high. This suggests that the true effect is likely to be close to the estimated effect, indicating a high probability that the study results are clinically relevant.

Several other meta-analyses reporting on the use of adipose-derived mesenchymal stem cells (ADMSCs) and bone marrow-derived mesenchymal stem cells (BMSCs) for the conservative treatment of knee osteoarthritis have been published over the past five years [1, 8, 11, 16, 19, 20, 30]. Of these seven studies, only one, by Dai et al. [31], did not demonstrate significant differences favouring mesenchymal stromal cells over other injection therapy modalities.

Kim et al. [10] included five studies published between 2013 and 2020, demonstrating an improvement in the WOMAC score. However, their analysis is limited by potential selection bias due to the inclusion of both autologous and allogeneic cells. Han et al. [32] included nine studies published between 2013 and 2019 and reported the superiority of mesenchymal stromal cells for the VAS but not the WOMAC score, with limitations arising from the inclusion of both autologous and allogeneic cells. Agarwal et al. [33] included 18 studies published between 2013 and 2020, showing significant differences favouring mesenchymal stromal cells in the WOMAC score. This study is severely limited by bias due to the inclusion of studies that also used arthroscopic intervention and stromal vascular fraction injections. Issa et al. [34] included only four studies, all published in 2019, and showed an improvement in the WOMAC score in favour of mesenchymal stromal cells. The included studies used only autologous mesenchymal cells, resulting in the lowest heterogeneity regarding the injected material among these meta-analyses. Kim et al. [10] included five studies published between 2019 and 2020, reporting an advantage of mesenchymal cells but no significant between-group differences. Their study is also limited by additional surgical interventions in three studies and the use of stromal vascular fraction in two studies. Finally, Muthu et al. [35] included 21 studies published between 2002 and 2020, showing that MSCs are superior to other interventions, resulting in less pain and better function. However, the authors included studies with additional surgical interventions, allogeneic cells, and stromal vascular fraction, which limits the conclusions drawn.

Dai et al. [31] included 13 studies published between 2015 and 2020 and could not demonstrate any differences between the intervention and control groups. Unfortunately, they also included studies applying allogeneic mesenchymal stromal cells. Interestingly, Dai et al. [31] demonstrated significant differences in favor of mesenchymal stromal cells (MSCs) for pain and WOMAC scores when compared to a control group or a control group using hyaluronic acid. However, they could not demonstrate significant differences in pain scores when comparing MSCs to a placebo. Therefore, one could argue that their study [31] also supports the use of MSC injections compared to other treatment modalities. The authors utilized the Minimal Clinically Important Difference (MCID) as a differentiator to assess the effect of the injections. They argued that despite achieving statistical significance, the differences did not reach the MCID threshold, indicating that the observed changes may not be clinically meaningful.

To minimize bias and enhance the validity of our findings, we have exclusively included studies that compared autologous and cultured adipose-derived and bone marrow-derived stromal cells with other orthobiologic interventions and injection therapies. Additionally, we have excluded studies older than ten years due to the rapid advancements in this field. The volume of publications has increased exponentially over the past decade, reflecting the swift progress in research and development [36]. Seventy percent of the included studies exhibited a low risk of bias, while two studies were categorized as high risk primarily due to confounding factors. The GRADE quality assessment resulted in an overall high to moderate level of certainty, suggesting that the confidence in the effect estimate is close to the true effect. Furthermore, the study quality assessment using the CMS score indicated that 70% of the studies were of high or good quality. Therefore, one can cautiously conclude that the results of this meta-analysis are both valid and clinically relevant. This conclusion is supported by the observation that study quality was primarily reduced due to a relatively short mean follow-up period of less than three years and insufficient description of post-intervention rehabilitation protocols. However, these variables may be less critical since a long-lasting effect of injections on symptom reduction in patients with knee osteoarthritis may not be anticipated. Many clinical studies are limited by small sample sizes, which may lead to Type II errors and undermine the validity of their findings. Katz et al. demonstrated that a minimum sample size of 172 patients is necessary to achieve a power of 0.9 [37]. This meta-analysis, which includes a total of 563 patients, enhances the statistical power and supports the potential clinical efficacy of mesenchymal stromal cells (MSCs) in alleviating pain in individuals with knee osteoarthritis.

Osteoarthritis is characterized by the irreversible degeneration of articular cartilage, which often results in significant functional impairment [38]. When conservative treatments fail, total knee arthroplasty becomes the primary surgical intervention [39]. In recent years, orthobiologics have emerged as promising alternatives, offering potential benefits such as pain reduction, functional enhancement, and the possibility for tissue repair and regeneration. Among these therapies, cell-based therapy with either adipose or bone marrow-derived stromal cells have shown promise in alleviating symptoms and improving both functional capacity and overall quality of life [9, 18, 23, 27, 28, 40].

Initially, it was thought that the therapeutic effects of mesenchymal stem cells (MSCs) were primarily due to their ability to migrate and engraft in targeted tissues [41]. However, subsequent research has demonstrated that the beneficial outcomes of MSCs are largely attributable to their secretion of trophic factors [41]. These factors play a crucial role in mediating cell-to-cell communication, regulating cell proliferation and differentiation, and exerting anti-inflammatory effects [41]. The paracrine effects of mesenchymal stromal cells (MSCs) reducing inflammatory responses may therefore be the primary mechanism of action contributing to the observed reduction in pain and improvement in function following MSC injections into the knee [38].

The results of this meta-analysis should be considered within the context of several limitations. The potential influence of missed studies cannot be ruled out, despite the use of comprehensive search strategies. These strategies were, however, restricted to publications in English and German, which may have resulted in the omission of high-quality evidence published in other languages. Unfortunately, the presence of publication bias was confirmed, which may be attributable to the exclusion of search strategies in other languages, particularly Chinese. We excluded one study published in Chinese, which suggests that other relevant studies may have been overlooked. Additionally, the asymmetry of the funnel plot and the significant result from Egger's test may partly be explained by the heterogeneity of the included studies, which could suggest that some effect sizes in the comparative analyses may be slightly overestimated. However, if overestimation is assumed, it would imply that MSC injection therapy may not be as effective for pain and further strengthening the conclusion that MSCs have no effect on clinical outcomes and function, as suggested by this study. The included studies were heterogeneous with respect to the source of cells used. Five studies employed bone marrow-derived stem cells (BMSCs), while five studies used adipose-derived stem cells (ADSCs). This variation in cell source may have influenced the results. Han et al. [32] suggested that ADSCs might be superior; however, their analysis reported a mean difference (MD) of 1.64 for BMSCs and 1.85 for ADSCs at six months, with similar MDs observed at twelve months. Despite the statistical significance of these findings, the clinical relevance of the treatment effects may therefore be limited. The dosage of injected mesenchymal stromal cells (MSCs) varied across studies, with some studies not specifying the dosages used. It is possible that higher doses of MSCs may have led to improved outcomes. Additionally, the control groups were heterogeneous, encompassing various treatment options, which could have introduced bias and potentially skewed the results in either direction. Other meta-analysis techniques, such as subgroup analysis, meta-regression, and sensitivity analysis, offer valuable methods for exploring potential sources of heterogeneity. However, these approaches require more than ten studies to yield reliable and valid results; without this threshold, their findings may be unstable or misleading. Unfortunately, our study was not sufficiently powered to apply these methods without introducing potential bias. Consequently, it is possible that the inclusion of additional studies could alter the treatment effect in either direction.

## Conclusions

The results of this meta-analysis demonstrated a significant effect of adipose and bone marrow-derived stromal cell (MSC) injections on pain reduction at all assessed time points, and MSC injections showed superiority over other non-surgical treatment options. These differences were not reflected in clinical and functional outcomes, indicating that the observed reduction in pain did not correspond to substantial improvements in knee functionality.

## Supplementary Information

Below is the link to the electronic supplementary material.

**Supplementary Figure 1:** PRISMA Flow Diagram. From the initial 1576 records, 10 studies were included in the quantitative synthesis. (TIF 66 kb)

**Supplementary Figure 2:** The funnel plot exhibited asymmetry in the standard deviations of the means, suggesting the presence of publication bias. (TIF 45 kb)

**Supplementary Figure 3:** The Forest Plot did not demonstrate significant between group differences at baseline. (TIF 35 kb)

**Supplementary Figure 4:** Forest Plot comparing VAS at 12 months demonstrated between group significant differences (p=0.001) in favor of MSCs. (TIF 28 kb)

**Supplementary Figure 5:** Forest Plot comparing the KOOS subscale pain at baseline demonstrated significant between group differences in favor of the control group (p=0.02) (TIF 26 kb)

**Supplementary Figure 6:** Forest Plot comparing the KOOS subscale symptoms at baseline did not demonstrate significant between group differences. (p=0.069) (TIF 29 kb)

**Supplementary Figure 7:** Forest Plot comparing the KOOS subscale ADL at baseline could not demonstrate significant between group differences (p=0.511) (TIF 29 kb)

**Supplementary Figure 8:** Forest Plot comparing the KOOS subscale sports at baseline could not demonstrate significant between group differences (p=0.298) (DOCX 34 kb)

**Supplementary Figure 9:** Forest Plot comparing the KOOS subscale QOL at baseline could not demonstrate significant between group differences (p=0.272) (TIF 47 kb)

**Supplementary Figure 10:** Forest Plot comparing the total WOMAC score at baseline could not demonstrate significant between group differences (p=0.215) (TIF 32 kb)

**Supplementary Figure 11:** Forest Plot comparing the WOMAC subscore pain at baseline did not demonstrate significant between group differences (p=0.169) (TIF 59 kb)

**Supplementary Figure 12:** Forest Plot comparing the WOMAC subscore pain at 3 months did not demonstrate significant between group differences (p=0.448) (TIF 53 kb)

**Supplementary Figure 13:** Forest Plot comparing the WOMAC sub-score pain at 6 months did not demonstrate significant between group differences (p=0.162) (DOCX 158 kb)

**Supplementary Figure 14:** Forest Plot comparing the WOMAC subscore pain at 12 months did not demonstrate significant between group differences (p=0.204) (DOCX 134 kb)

**Supplementary Figure 15:** Forest Plot comparing the WOMAC subscore stiffness at baseline did not demonstrate significant between group differences (p=0.119) (TIF 154 kb)

**Supplementary Figure 16:** Forest Plot comparing the WOMAC subscore stiffness at 3 months demonstrated near significant between group differences in favor of MSCs (p=0.072) (TIF 54 kb)

**Supplementary Figure 17:** Forest Plot comparing the WOMAC subscore stiffness at 6 months did not demonstrate significant between group differences (p=0.678) (TIF 62 kb)

**Supplementary Figure 18:** Forest Plot comparing the WOMAC subscore stiffness at 12 months did not demonstrate significant between group differences (p=0.856) (TIF 54 kb)

**Supplementary Figure 19:** Forest Plot comparing the WOMAC subscore function at baseline did not demonstrate significant between group differences (p=0.082) (TIF 57 kb)

**Supplementary Figure 20:** Forest Plot comparing the WOMAC subscore function at 3 months did not demonstrate significant between group differences (p=0.946) (TIF 59 kb)

**Supplementary Figure 21:** Forest Plot comparing the WOMAC subscore function at 6 months did not demonstrate significant between group differences (p=0.112) (TIF 59 kb)

**Supplementary Figure 22:** Forest Plot comparing the WOMAC subscore function at 12 months did not demonstrate significant between group differences (p=0.488) (TIF 49 kb)

## Data Availability

No datasets were generated or analysed during the current study.
