# Supplementary figures and images for: Adipose- and bone marrow-derived stromal cells reduce pain in patients with knee osteoarthritis but do not substantially improve knee functionality: an updated systematic review and meta-analysis
Source: Eur J Orthop Surg Traumatol. 2025 May 23;35(1):214. doi: 10.1007/s00590-025-04322-4 (PMC12102120; doi:10.1007/s00590-025-04322-4)

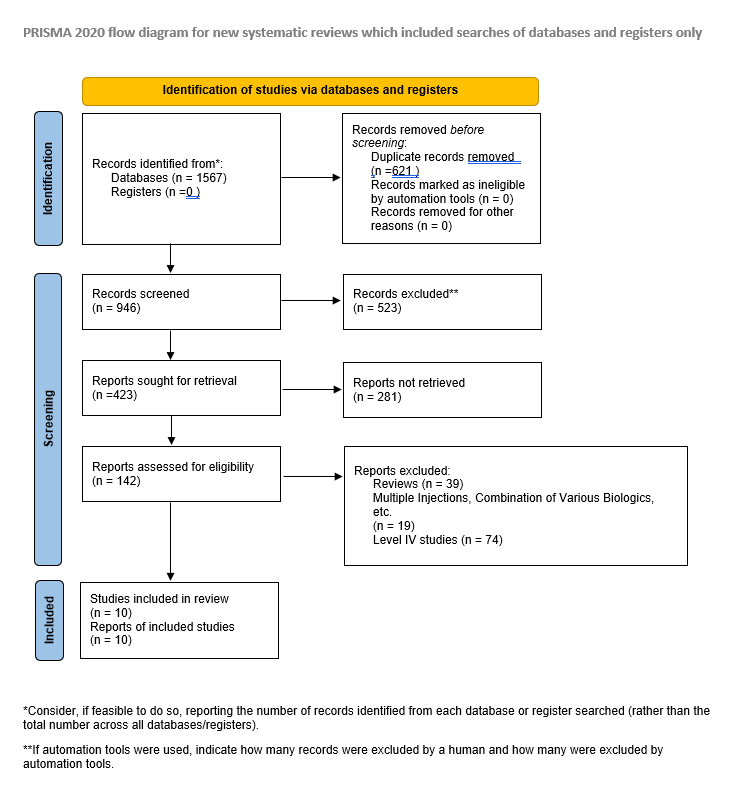

Supplement: Supplementary file 1 — Supplementary Figure 1: PRISMA Flow Diagram. From the initial 1576 records, 10 studies were included in the quantitative synthesis. (TIF 66 kb) [file 590_2025_4322_MOESM1_ESM.tif]

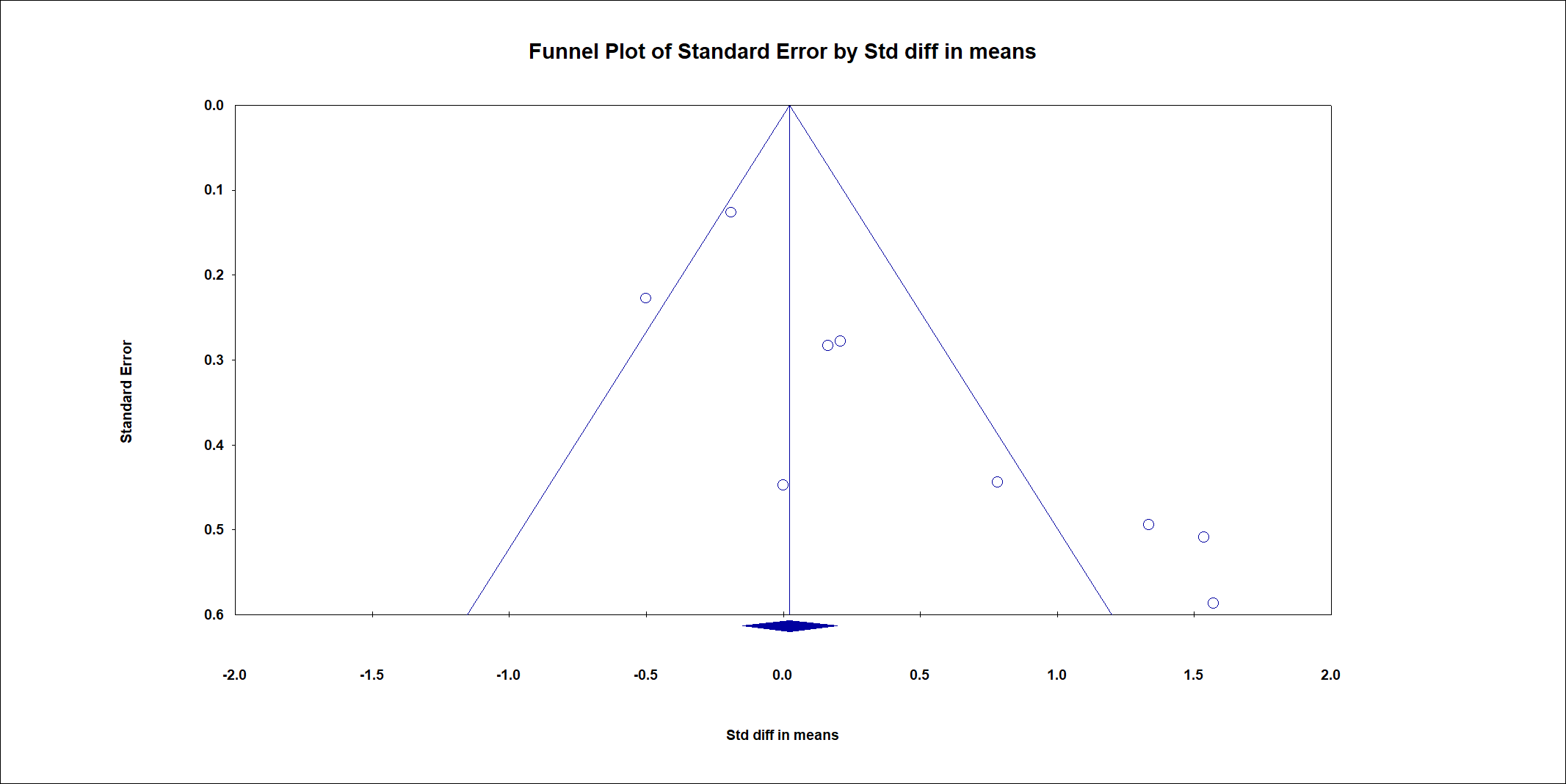

Supplement: Supplementary file 2 — Supplementary Figure 2: The funnel plot exhibited asymmetry in the standard deviations of the means, suggesting the presence of publication bias. (TIF 45 kb) [file 590_2025_4322_MOESM2_ESM.tif]

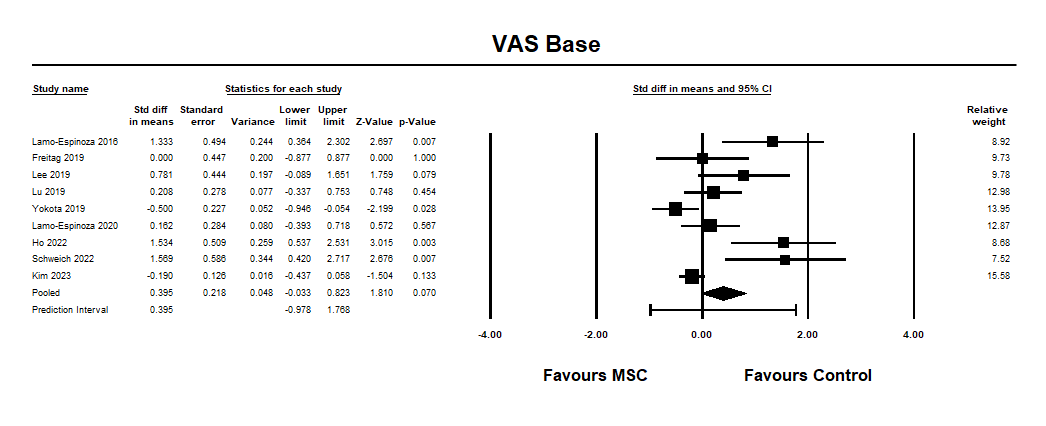

Supplement: Supplementary file 3 — Supplementary Figure 3: The Forest Plot did not demonstrate significant between group differences at baseline. (TIF 35 kb) [file 590_2025_4322_MOESM3_ESM.tif]

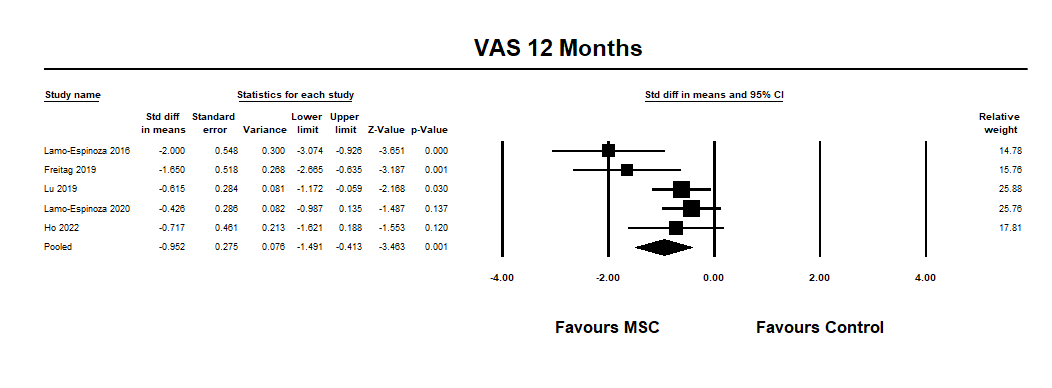

Supplement: Supplementary file 4 — Supplementary Figure 4: Forest Plot comparing VAS at 12 months demonstrated between group significant differences (p=0.001) in favor of MSCs. (TIF 28 kb) [file 590_2025_4322_MOESM4_ESM.tif]

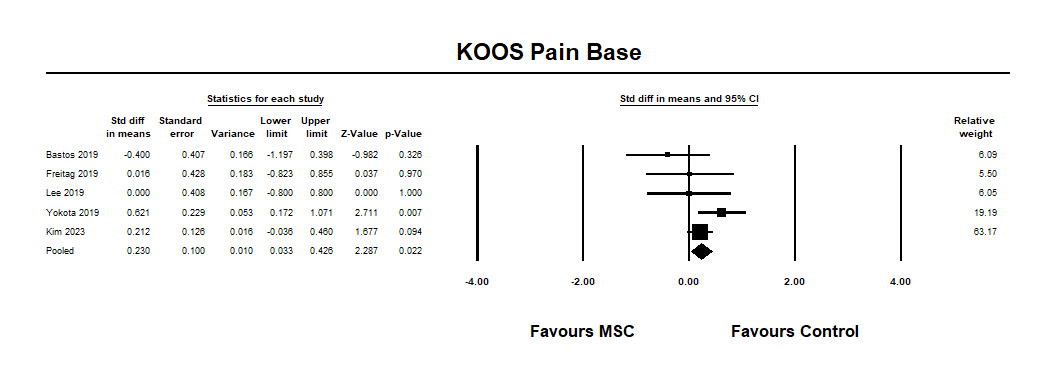

Supplement: Supplementary file 5 — Supplementary Figure 5: Forest Plot comparing the KOOS subscale pain at baseline demonstrated significant between group differences in favor of the control group (p=0.02) (TIF 26 kb) [file 590_2025_4322_MOESM5_ESM.tif]

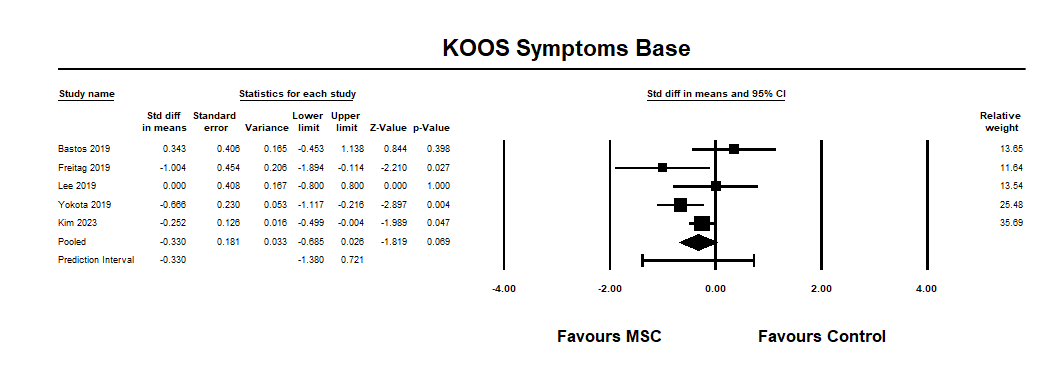

Supplement: Supplementary file 6 — Supplementary Figure 6: Forest Plot comparing the KOOS subscale symptoms at baseline did not demonstrate significant between group differences. (p=0.069) (TIF 29 kb) [file 590_2025_4322_MOESM6_ESM.tif]

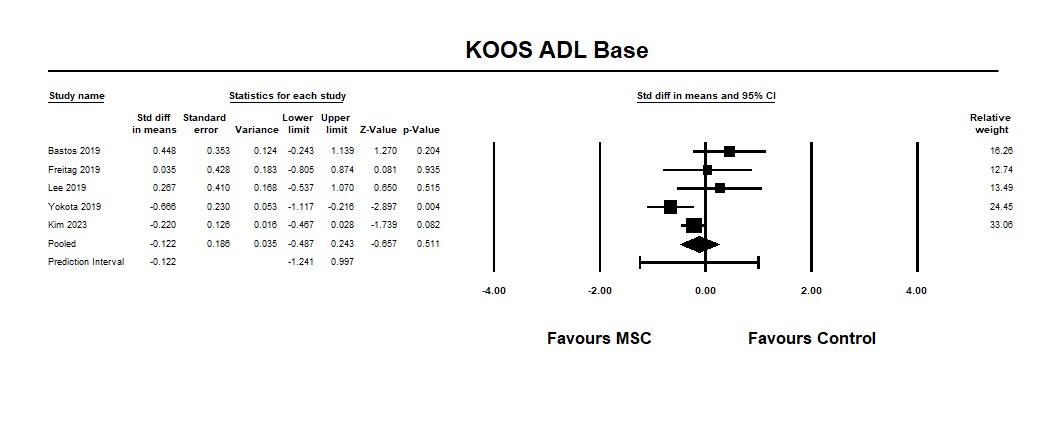

Supplement: Supplementary file 7 — Supplementary Figure 7: Forest Plot comparing the KOOS subscale ADL at baseline could not demonstrate significant between group differences (p=0.511) (TIF 29 kb) [file 590_2025_4322_MOESM7_ESM.tif]

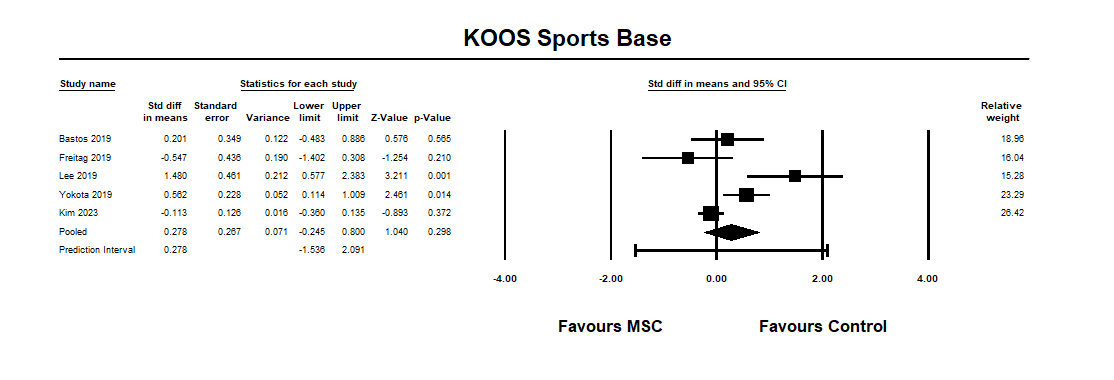

Supplement: Supplementary file 8 — Supplementary Figure 8: Forest Plot comparing the KOOS subscale sports at baseline could not demonstrate significant between group differences (p=0.298) (DOCX 34 kb) [file 590_2025_4322_MOESM8_ESM.docx]

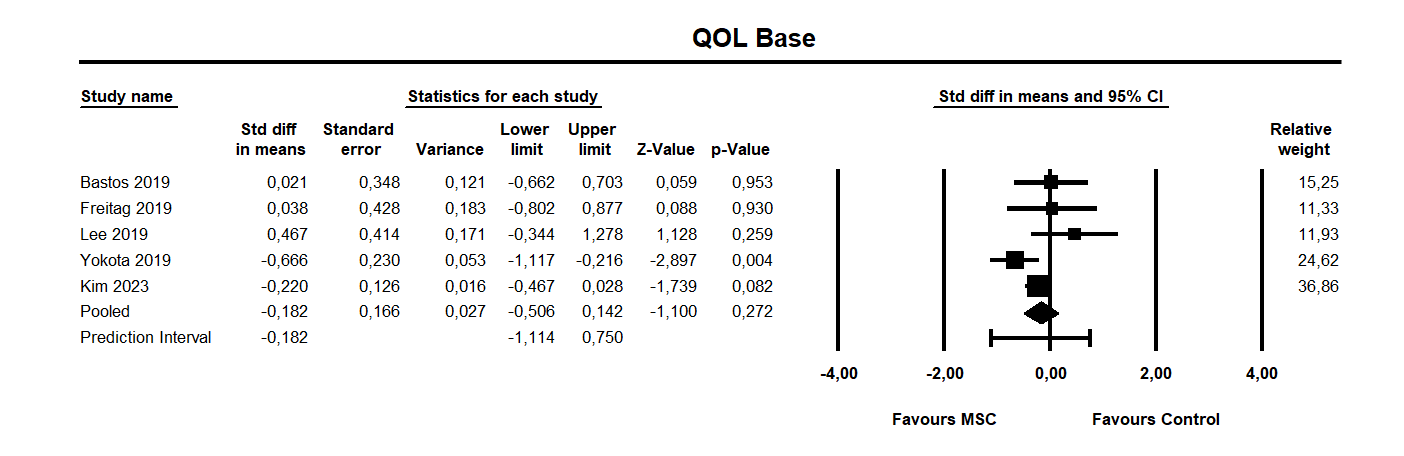

Supplement: Supplementary file 9 — Supplementary Figure 9: Forest Plot comparing the KOOS subscale QOL at baseline could not demonstrate significant between group differences (p=0.272) (TIF 47 kb) [file 590_2025_4322_MOESM9_ESM.tif]

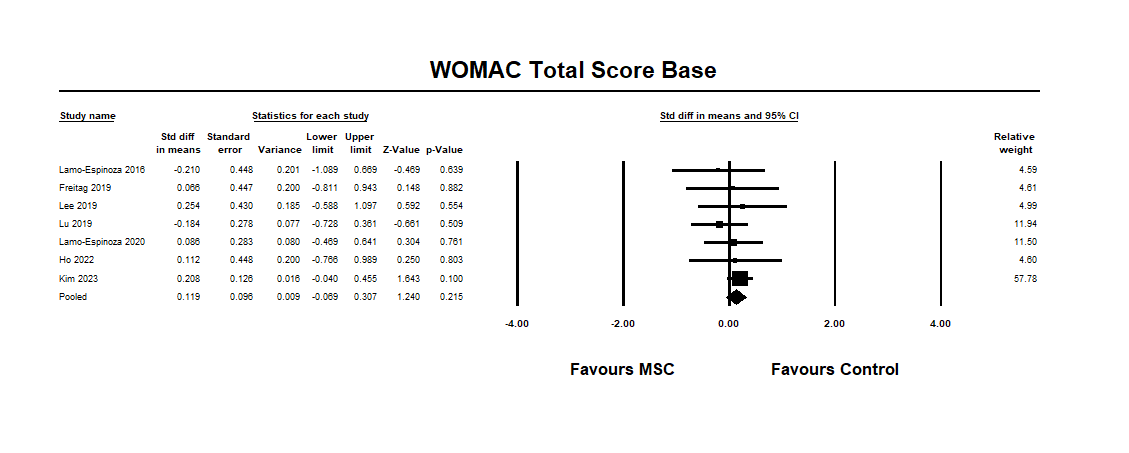

Supplement: Supplementary file 10 — Supplementary Figure 10: Forest Plot comparing the total WOMAC score at baseline could not demonstrate significant between group differences (p=0.215) (TIF 32 kb) [file 590_2025_4322_MOESM10_ESM.tif]

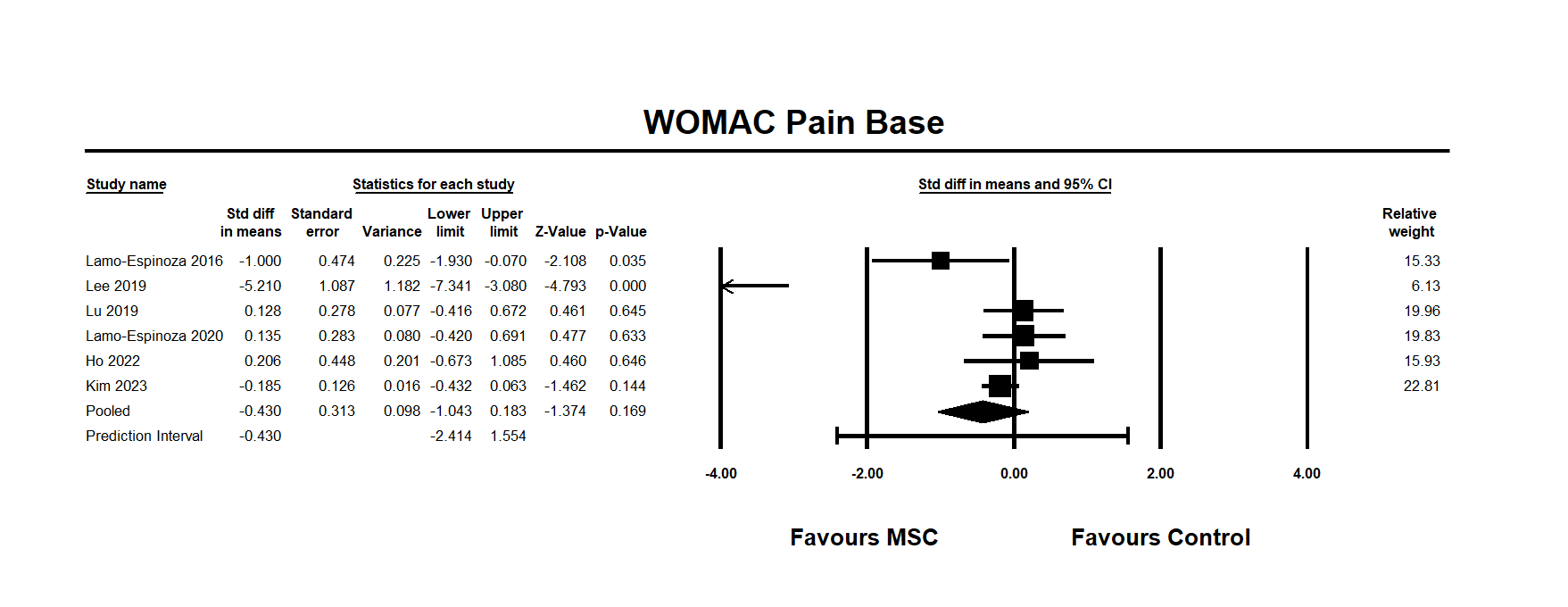

Supplement: Supplementary file 11 — Supplementary Figure 11: Forest Plot comparing the WOMAC subscore pain at baseline did not demonstrate significant between group differences (p=0.169) (TIF 59 kb) [file 590_2025_4322_MOESM11_ESM.tif]

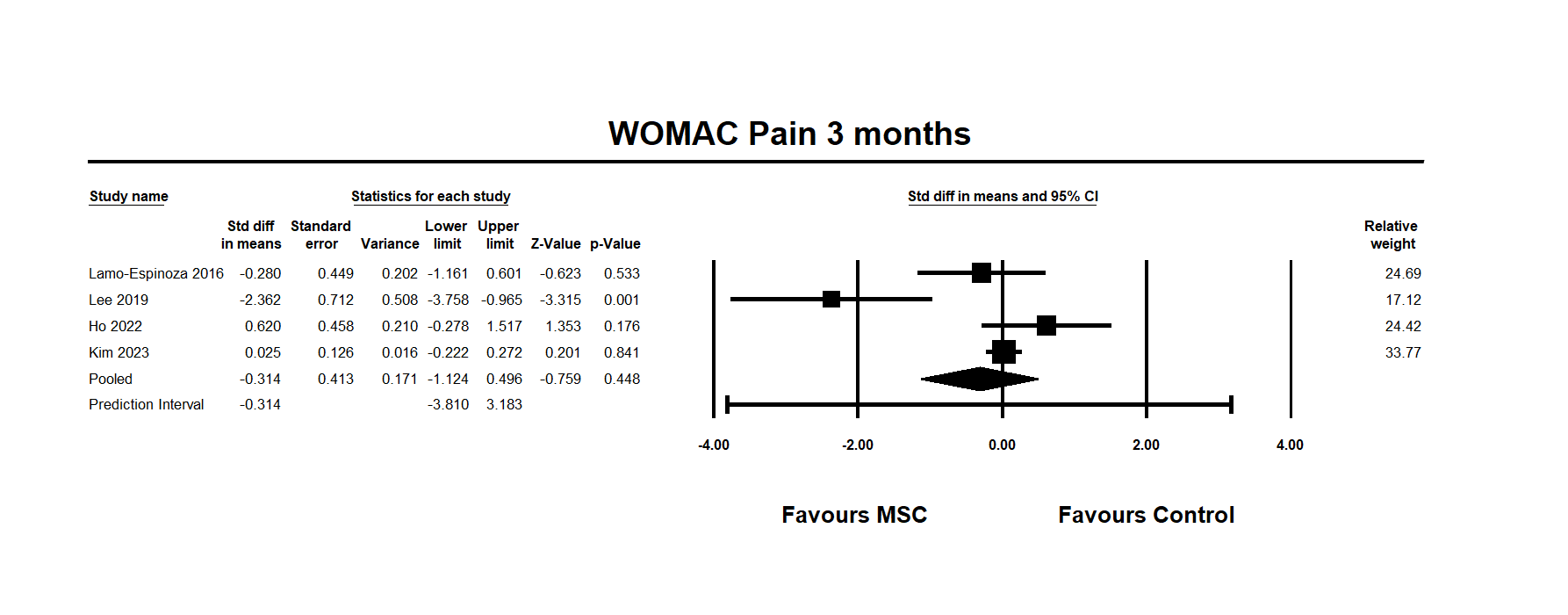

Supplement: Supplementary file 12 — Supplementary Figure 12: Forest Plot comparing the WOMAC subscore pain at 3 months did not demonstrate significant between group differences (p=0.448) (TIF 53 kb) [file 590_2025_4322_MOESM12_ESM.tif]

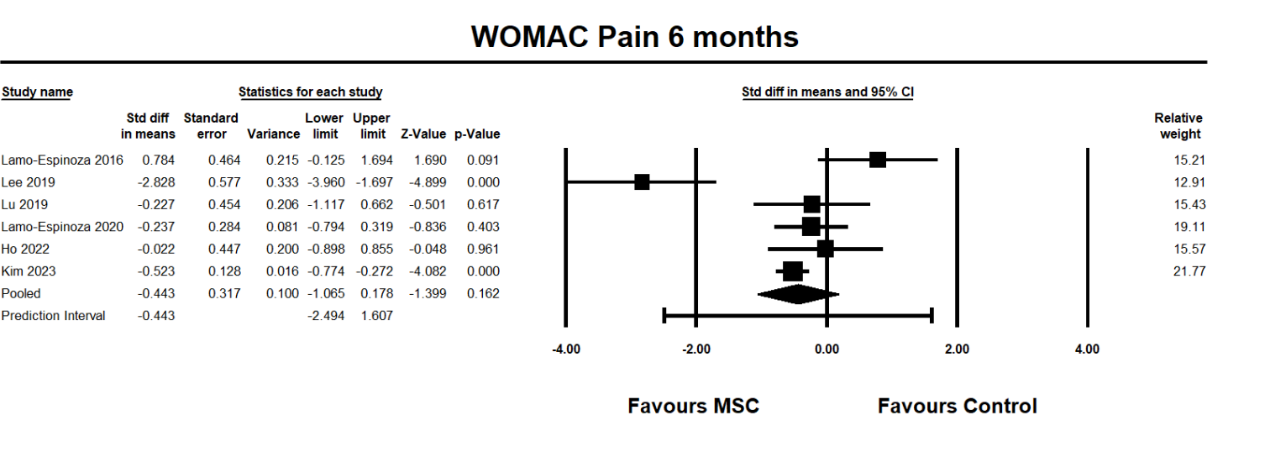

Supplement: Supplementary file 13 — Supplementary Figure 13: Forest Plot comparing the WOMAC sub-score pain at 6 months did not demonstrate significant between group differences (p=0.162) (DOCX 158 kb) [file 590_2025_4322_MOESM13_ESM.docx]

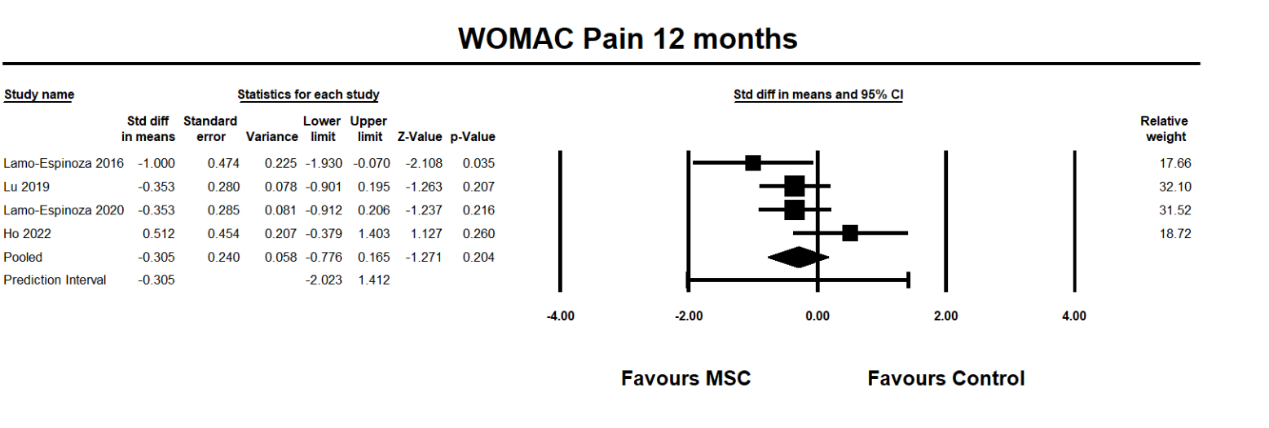

Supplement: Supplementary file 14 — Supplementary Figure 14: Forest Plot comparing the WOMAC subscore pain at 12 months did not demonstrate significant between group differences (p=0.204) (DOCX 134 kb) [file 590_2025_4322_MOESM14_ESM.docx]

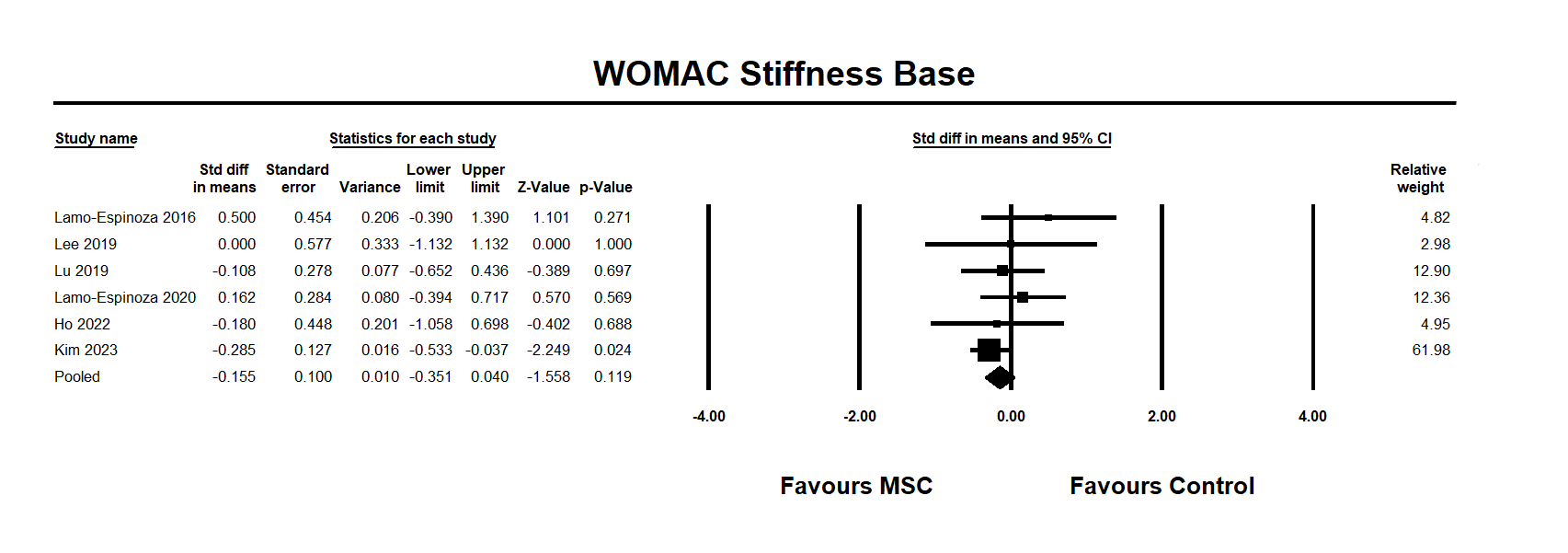

Supplement: Supplementary file 15 — Supplementary Figure 15: Forest Plot comparing the WOMAC subscore stiffness at baseline did not demonstrate significant between group differences (p=0.119) (TIF 154 kb) [file 590_2025_4322_MOESM15_ESM.tif]

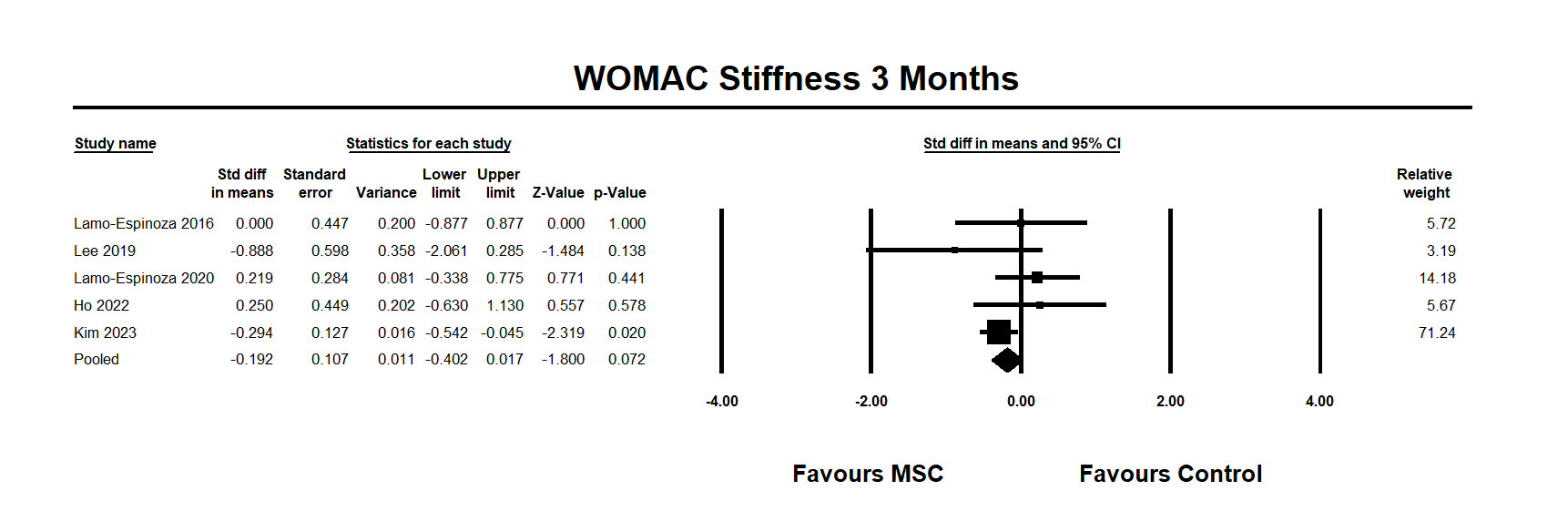

Supplement: Supplementary file 16 — Supplementary Figure 16: Forest Plot comparing the WOMAC subscore stiffness at 3 months demonstrated near significant between group differences in favor of MSCs (p=0.072) (TIF 54 kb) [file 590_2025_4322_MOESM16_ESM.tif]

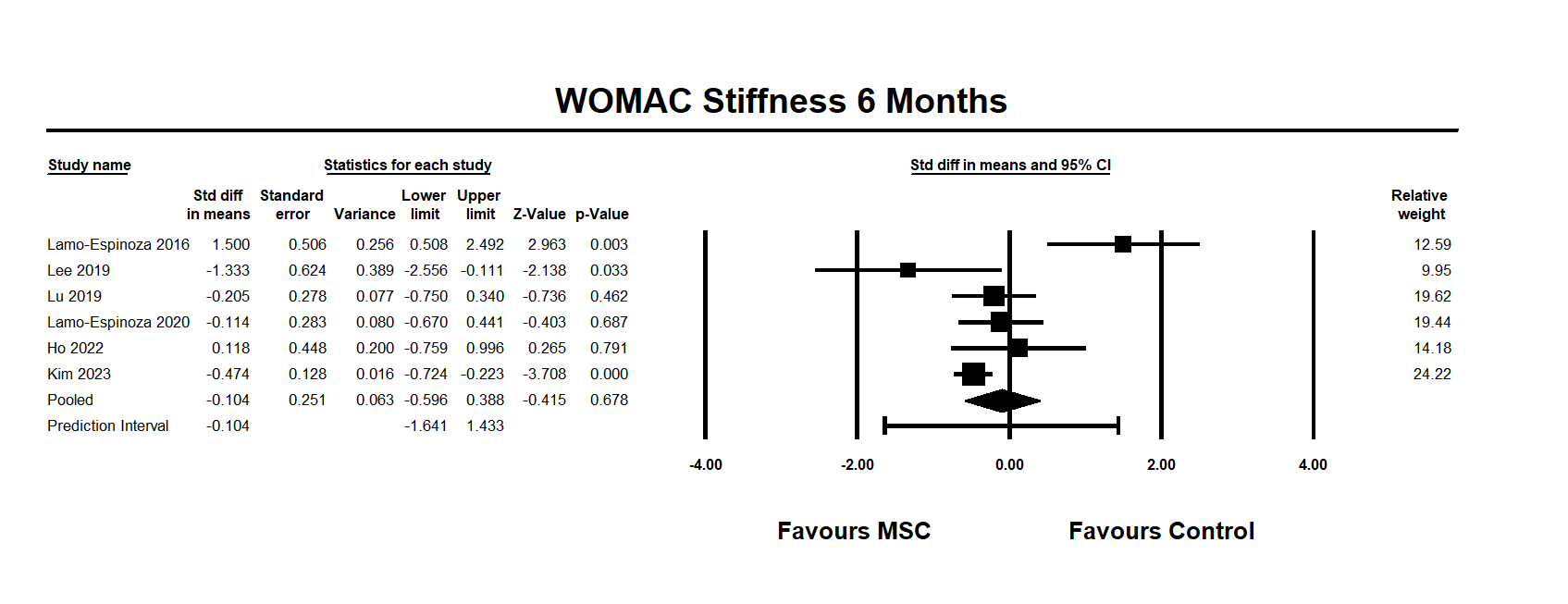

Supplement: Supplementary file 17 — Supplementary Figure 17: Forest Plot comparing the WOMAC subscore stiffness at 6 months did not demonstrate significant between group differences (p=0.678) (TIF 62 kb) [file 590_2025_4322_MOESM17_ESM.tif]

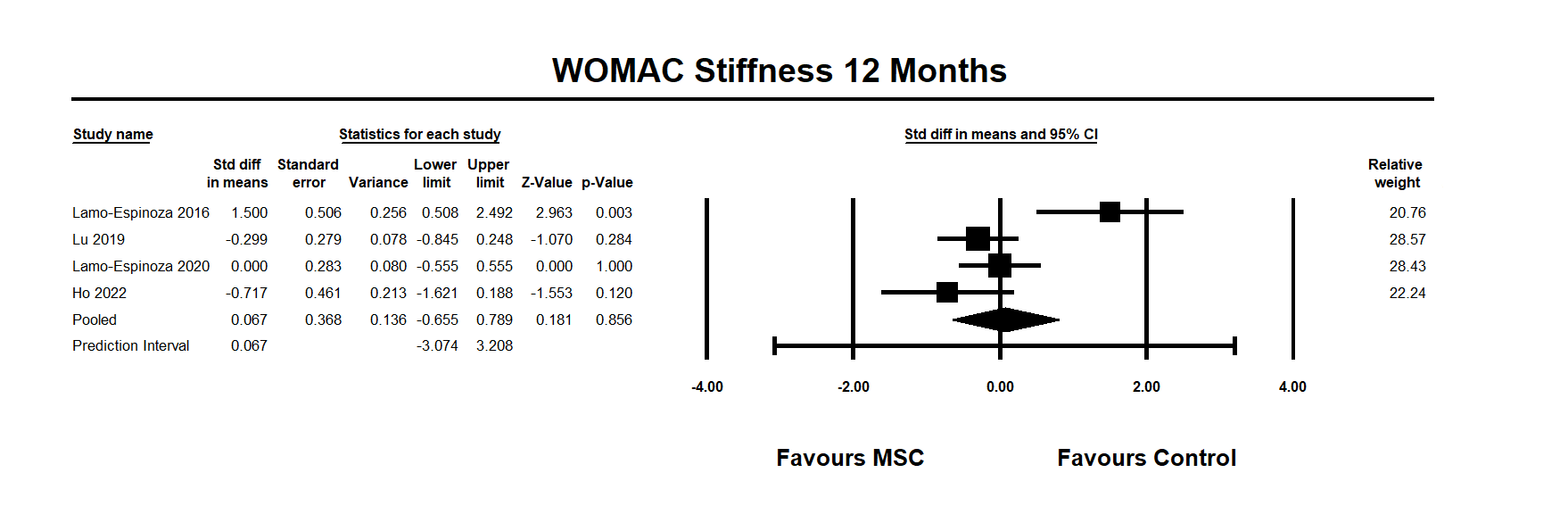

Supplement: Supplementary file 18 — Supplementary Figure 18: Forest Plot comparing the WOMAC subscore stiffness at 12 months did not demonstrate significant between group differences (p=0.856) (TIF 54 kb) [file 590_2025_4322_MOESM18_ESM.tif]

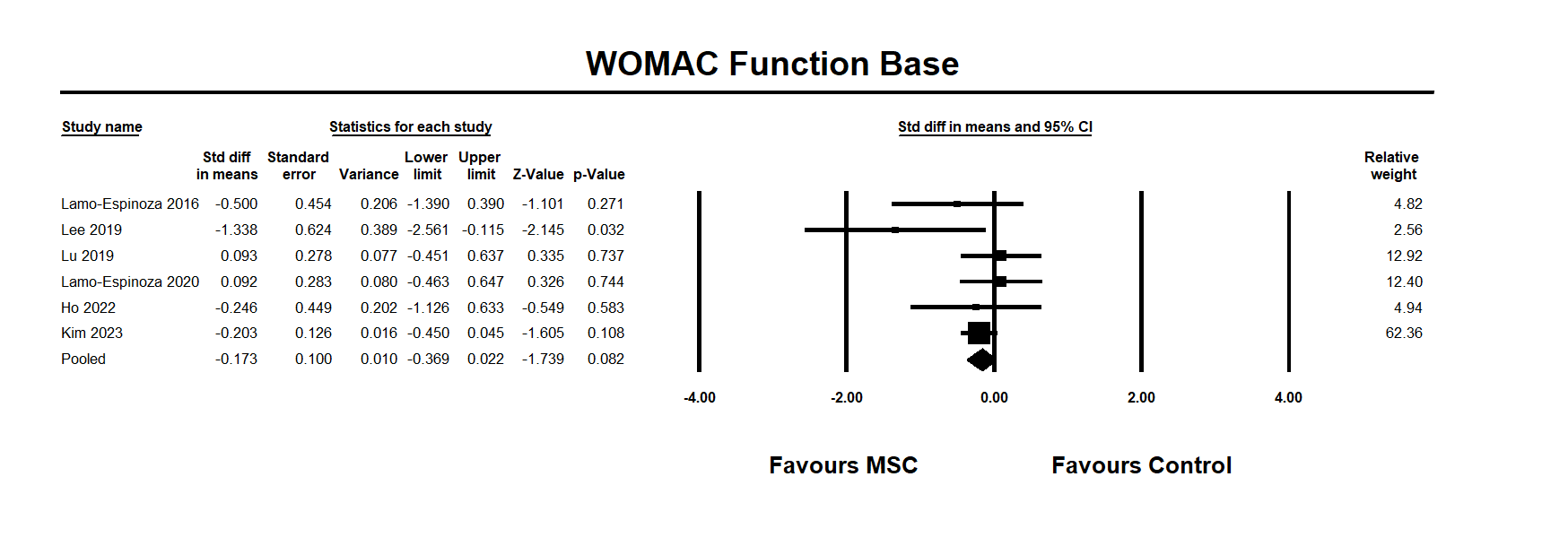

Supplement: Supplementary file 19 — Supplementary Figure 19: Forest Plot comparing the WOMAC subscore function at baseline did not demonstrate significant between group differences (p=0.082) (TIF 57 kb) [file 590_2025_4322_MOESM19_ESM.tif]

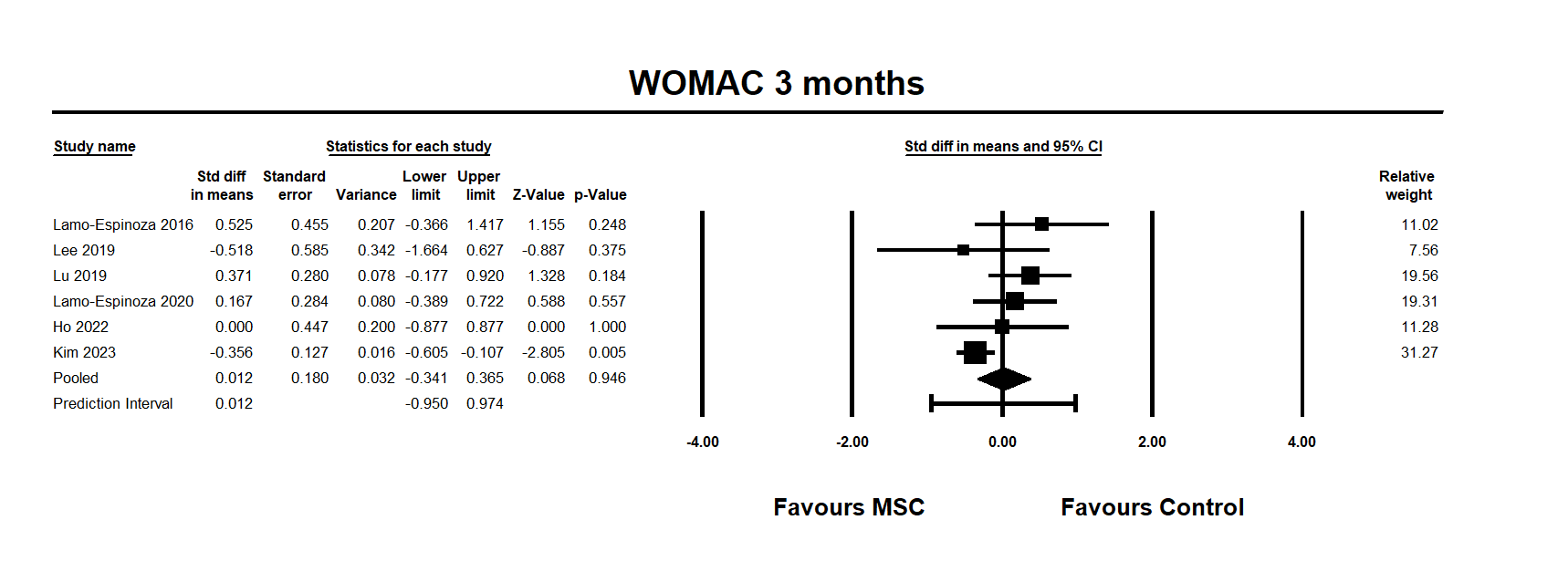

Supplement: Supplementary file 20 — Supplementary Figure 20: Forest Plot comparing the WOMAC subscore function at 3 months did not demonstrate significant between group differences (p=0.946) (TIF 59 kb) [file 590_2025_4322_MOESM20_ESM.tif]

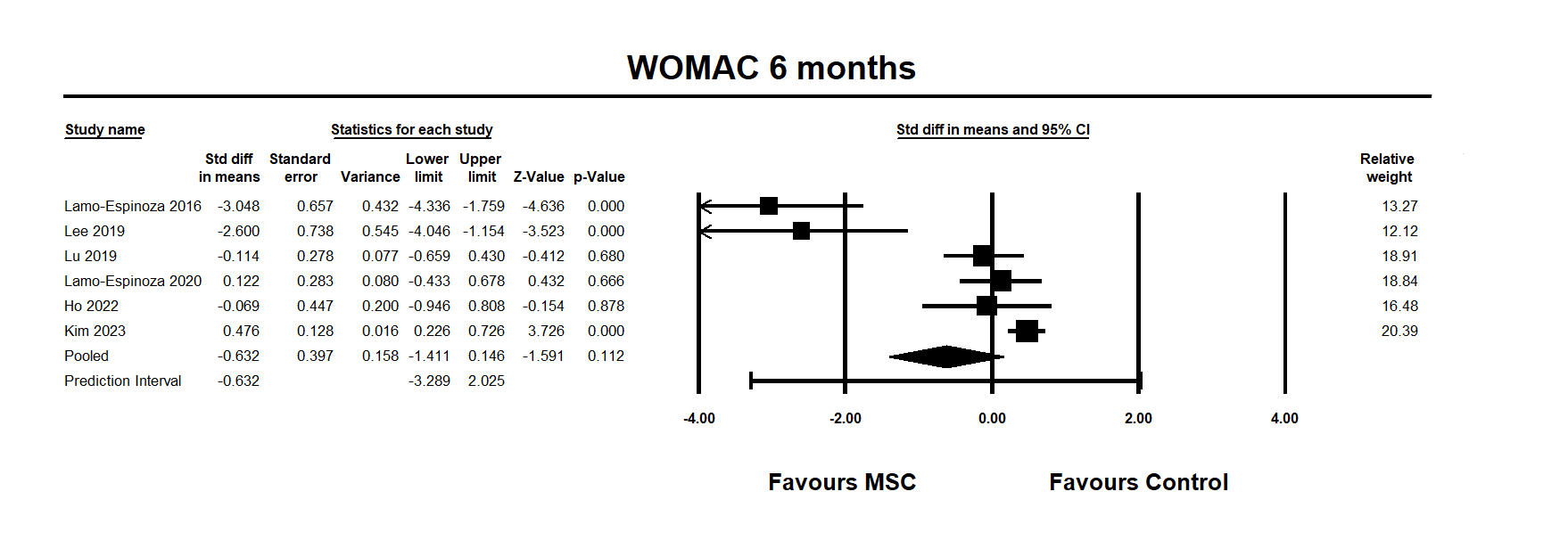

Supplement: Supplementary file 21 — Supplementary Figure 21: Forest Plot comparing the WOMAC subscore function at 6 months did not demonstrate significant between group differences (p=0.112) (TIF 59 kb) [file 590_2025_4322_MOESM21_ESM.tif]

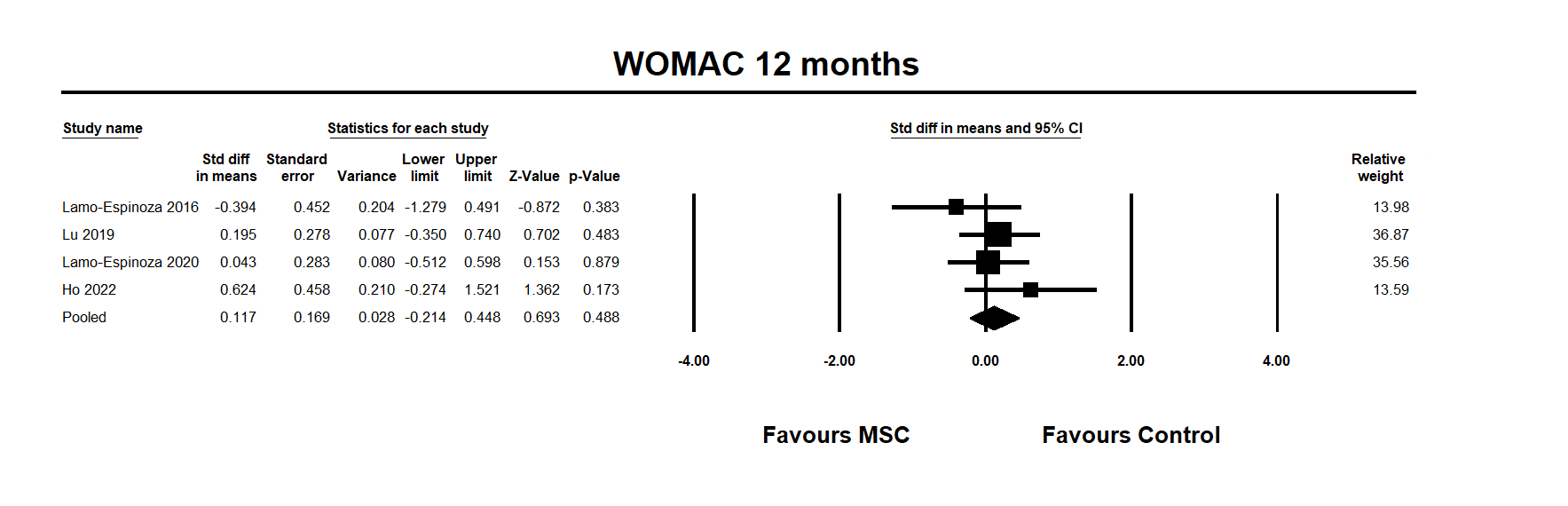

Supplement: Supplementary file 22 — Supplementary Figure 22: Forest Plot comparing the WOMAC subscore function at 12 months did not demonstrate significant between group differences (p=0.488) (TIF 49 kb) [file 590_2025_4322_MOESM22_ESM.tif]
